# A Unique *Saccharomyces cerevisiae* × *Saccharomyces uvarum* Hybrid Isolated From Norwegian Farmhouse Beer: Characterization and Reconstruction

**DOI:** 10.3389/fmicb.2018.02253

**Published:** 2018-09-24

**Authors:** Kristoffer Krogerus, Richard Preiss, Brian Gibson

**Affiliations:** ^1^VTT Technical Research Centre of Finland Ltd., Espoo, Finland; ^2^Department of Biotechnology and Chemical Technology, School of Chemical Technology, Aalto University, Espoo, Finland; ^3^Department of Molecular and Cellular Biology, University of Guelph, Guelph, ON, Canada; ^4^Escarpment Laboratories, Guelph, ON, Canada

**Keywords:** yeast, beer, hybrid, kveik, dextrin, genome

## Abstract

An unknown interspecies *Saccharomyces* hybrid, “Muri,” was recently isolated from a “kveik” culture, a traditional Norwegian farmhouse brewing yeast culture (Preiss et al., 2018). Here we used whole genome sequencing to reveal the strain as an allodiploid *Saccharomyces cerevisiae* × *Saccharomyces uvarum* hybrid. Phylogenetic analysis of its sub-genomes revealed that the *S. cerevisiae* and *S. uvarum* parent strains of Muri appear to be most closely related to English ale and Central European cider and wine strains, respectively. We then performed phenotypic analysis on a number of brewing-relevant traits in a range of *S. cerevisiae*, *S. uvarum* and hybrid strains closely related to the Muri hybrid. The Muri strain possesses a range of industrially desirable phenotypic properties, including broad temperature tolerance, good ethanol tolerance, and efficient carbohydrate use, therefore making it an interesting candidate for not only brewing applications, but potentially various other industrial fermentations, such as biofuel production and distilling. We identified the two *S. cerevisiae* and *S. uvarum* strains that were genetically and phenotypically most similar to the Muri hybrid, and then attempted to reconstruct the Muri hybrid by generating *de novo* interspecific hybrids between these two strains. The *de novo* hybrids were compared with the original Muri hybrid, and many appeared phenotypically more similar to Muri than either of the parent strains. This study introduces a novel approach to studying hybrid strains and strain development by combining genomic and phenotypic analysis to identify closely related parent strains for construction of *de novo* hybrids.

## Introduction

Yeast has a central role in beer production and is responsible for the conversion of wort carbohydrates into ethanol and CO_2_, as well as the synthesis of flavor compounds. Beer is traditionally fermented with domesticated strains of *Saccharomyces cerevisiae* and *Saccharomyces pastorianus* (Gibson et al., 2017). The demand from consumers for craft and specialty beers that have rich and unique aroma has increased in recent years (Aquilani et al., 2015), which has led breweries to explore alternative yeasts. One such group of yeasts that have recently gained attention are “kveik” yeasts: a range of traditional yeast cultures that have been used and maintained by Norwegian farmhouse brewers (Garshol, 2013, 2016; Fowle, 2017; Preiss et al., 2018). The genetic and phenotypic diversity of a range of kveik strains was recently explored by Preiss et al. (2018). The vast majority of the isolates were identified as strains of *S. cerevisiae*, which formed a genetically distinct group possessing properties relevant to brewing. One isolate, however, was found to be an unknown *Saccharomyces* interspecies hybrid. Here, we aimed to identify and characterize this hybrid strain.

The use of interspecific yeast hybrids for beer fermentation is widespread, with lager yeast, i.e., *S. pastorianus*, being used for the majority of global beer production. This *S. cerevisiae* × *Saccharomyces eubayanus* hybrid combines the efficient wort sugar utilization of the *S. cerevisiae* parent with the cold tolerance of the *S. eubayanus* parent (Gibson and Liti, 2015). Interspecific hybridization not only allows for the combination of phenotypic traits from diverse parent strains, but hybrids often exhibit superior phenotypic qualities relative to parent strains, i.e., heterosis or hybrid vigor. The relatively harsh and stressful environment that yeast is exposed to during beer fermentation may have selected for interspecific hybrids, which have been shown to exhibit increased stress tolerance (Lopandic, 2018). In addition to the lager yeast, several other natural interspecific *Saccharomyces* hybrids have been isolated in brewing environments. Hybrids between *S. cerevisiae* and *Saccharomyces kudriavzevii* are used for the fermentation of several Belgian Trappist beers (González et al., 2008), while *Saccharomyces bayanus* (*S. eubayanus* × *S. uvarum*) hybrids have been isolated as contaminants from beer (Rainieri et al., 2006; Nguyen et al., 2011). Natural hybrids between *S. cerevisiae* and *S. uvarum* are also used in winemaking (Christine et al., 2007), but limited reports exist describing use of such hybrids in brewing.

In addition to natural *Saccharomyces* hybrids, *de novo* interspecific *Saccharomyces* hybrids can also readily be generated. These have been studied for their potential in a range of industrial applications, including biofuel production (Snoek et al., 2015; Peris et al., 2017), brewing (Krogerus et al., 2015; Mertens et al., 2015) and winemaking (Bellon et al., 2011; Origone et al., 2018). *De novo* hybrids have exhibited various improved traits compared to their parent strains, including faster fermentation rates, more complete sugar use, greater stress tolerance, and increases in aroma compound production (Bellon et al., 2011; Dunn et al., 2013; Steensels et al., 2014a; Krogerus et al., 2015; Mertens et al., 2015; Snoek et al., 2015). For brewing, much of the recent research has focused on the generation and characterization of new lager yeast, i.e., *S. cerevisiae* × *S. eubayanus*, hybrids (Hebly et al., 2015; Krogerus et al., 2015, 2016, 2017, 2018; Mertens et al., 2015; Alexander et al., 2016). However, alternative *Saccharomyces* interspecific hybrid combinations have also shown promise in brewing conditions (Sato et al., 2002; Nikulin et al., 2018). In addition to their industrial applications, *de novo* hybrids have also acted as useful models for studying adaptation and molding of hybrid genomes (Dunn et al., 2013; Peris et al., 2017; Smukowski Heil et al., 2017; Krogerus et al., 2018). Such studies could be useful for lager yeast in particular, as much of their natural evolutionary history still remains obscure despite their industrial importance (Baker et al., 2015; Okuno et al., 2015).

Recent whole genome sequencing studies have revealed multiple domestication events for *S. cerevisiae* (Gallone et al., 2016; Gonçalves et al., 2016; Peter et al., 2018). Commercially used brewing strains, for example, tend to cluster into one of two independently domesticated ‘Beer’ groups. While these studies have focused on *S. cerevisiae*, and limited data on interspecific *Saccharomyces* hybrids is available, Gonçalves et al. (2016) also revealed that the *S. cerevisiae* sub-genomes of lager yeasts group among the “Beer 1” or “Ale beer” yeasts. However, no single strain has yet been identified as the potential *S. cerevisiae* ale parent of lager yeast (Monerawela and Bond, 2018). In addition to providing valuable data on the diversity and history of brewing strains, whole genome sequence data, in combination with comprehensive phenotype data, are valuable resources for parent selection during breeding and hybridization. Gallone et al. (2016) demonstrated how a strain lacking phenolic off-flavor (POF) formation could be obtained through mating of parent strains carrying heterozygous loss-of-function polymorphisms in *FDC1*. Heterosis has also been shown to be positively correlated with sequence divergence during breeding of domesticated strains (Plech et al., 2014).

Recently, Preiss et al. (2018) described the isolation of an unknown *Saccharomyces* interspecies hybrid, i.e., the Muri strain, from a Norwegian farmhouse brewing (kveik) culture. This strain was identified as a *S. bayanus*-type hybrid based on internally transcribed spacer (ITS) sequencing. Here we aimed to identify, characterize, and ultimately reconstruct this hybrid. Whole genome sequencing was used to reveal the strain as an allodiploid *S. cerevisiae* × *S. uvarum* hybrid. We then performed phylogenetic analysis of its sub-genomes, in an attempt to identify *S. cerevisiae* and *S. uvarum* strains closely related to the parent strains of Muri. In addition, we compared a number of brewing-relevant phenotypic traits in *S. cerevisiae*, *S. uvarum* and hybrid strains closely related to the Muri hybrid. These data were used to identify two *S. cerevisiae* and *S. uvarum* strains that were genetically and phenotypically most similar to the Muri hybrid. We then attempted to reconstruct the Muri hybrid by generating *de novo* interspecific hybrids between these two strains. The *de novo* hybrids were compared with the original Muri hybrid, and appeared phenotypically more similar to Muri than either of the parent strains. This study introduces a novel approach to studying hybrid strains and strain development by combining genomic and phenotypic analysis to identify closely related parent strains for construction of *de novo* hybrids.

## Materials and Methods

### Yeast Strains

A list of strains used in this study can be found in **Table 1**. The *de novo* yeast hybrids between *S. cerevisiae* A241 and *S. uvarum* C995 were constructed by spore-to-spore mating as described previously (Krogerus et al., 2016). Potential hybrids were first identified by the ability to both grow at 37°C (*S. cerevisiae*-specific) and form blue-colored colonies (*S. uvarum*-specific) when grown in the presence of X-α-Gal (#16555 Sigma-Aldrich). Single cell isolates of potential hybrids were obtained by re-streaking single colonies three times on YP-Glucose agar. Hybrid status was confirmed through PCR using species-specific (*S. cerevisiae* and *S. uvarum*) primers (Muir et al., 2011; Pengelly and Wheals, 2013) and ITS-PCR followed by *HaeIII* digestion (Pham et al., 2011). The ploidy of the Muri strain was determined by flow cytometry as described previously (Krogerus et al., 2017).

**Table 1:** Yeast strains used in the study.

Code	Alternative name	Species	Origin	Reference
Muri		*S. cerevisiae* × *S. uvarum*	Beer, Norway	Preiss et al., 2018
A240	Beer032	*S. cerevisiae*	Beer, England	Gallone et al., 2016
A241	Beer059	*S. cerevisiae*	Beer, England	Gallone et al., 2016
A242	Beer062	*S. cerevisiae*	Beer, England	Gallone et al., 2016
A243	Beer091	*S. cerevisiae*	Beer, Germany	Gallone et al., 2016
C992	ZP556	*S. uvarum*	Oak tree, Canada	Almeida et al., 2014
C993	NCAIM 01116	*S. uvarum*	Grapes, Russia	Almeida et al., 2014
C995	ZP646	*S. uvarum*	Cider, Germany	Almeida et al., 2014
C996	CBS8614	*S. cerevisiae* × *S. kudriavzevii* × *S. uvarum*	Cider, France	Almeida et al., 2014
C997	CBS8615	*S. cerevisiae* × *S. uvarum*	Wine, Italy	Almeida et al., 2014
C998	NCAIM 00676	*S. eubayanus* × *S. uvarum*	Fermented beverage, Hungary	Almeida et al., 2014
3B	A241 × C995 3B	*S. cerevisiae* × *S. uvarum*	*de novo* hybrid	This study
4B	A241 × C995 4B	*S. cerevisiae* × *S. uvarum*	*de novo* hybrid	This study
6A	A241 × C995 6A	*S. cerevisiae* × *S. uvarum*	*de novo* hybrid	This study
13C	A241 × C995 13C	*S. cerevisiae* × *S. uvarum*	*de novo* hybrid	This study


### Growth Assays

Growth of the yeast strains at various temperatures (4, 12, 37, and 40 °C) was tested on YP-Glucose agar plates. Overnight pre-cultures of all the strains were grown in YP-Glucose at 25°C. The yeast was then pelleted and resuspended in 50 mM citrate buffer (pH 7.2) to deflocculate the yeast. The cell concentration was measured with a NucleoCounter YC-100 (ChemoMetec, Denmark), after which suspensions were diluted to contain approximately 10^5^, 10^4^, and 10^3^ cells ml^-1^. Five-microliter aliquots of the suspensions of each strain were spotted onto agar plates. Plates were sealed with Parafilm and incubated for up to 21 days, after which growth was scored based on colony size. Growth in the presence of ethanol was tested in YP-Glucose media supplemented with 11% ethanol. One-microliter of media was inoculated with the overnight pre-cultures to a starting OD600 of 0.05. Cultures were incubated at 25°C for 4 days, after which the optical densities of the cultures were measured.

### 100 mL-Scale Wort Fermentations

100 mL-scale fermentations were carried out in 250 mL Erlenmeyer flasks capped with glycerol-filled airlocks. Yeast strains were grown overnight in 50 mL YP-Maltose at 24°C. The pre-cultured yeast was then inoculated into 100 mL of 15°P all-malt wort at a rate of 10 × 10^6^ viable cells mL^-1^. Fermentations were carried out in duplicate at 12 and 20°C for 16 and 10 days, respectively, and these were monitored daily by mass lost as CO_2_. Samples for sugar, ethanol, and yeast-derived flavor compounds analysis were drawn from the beer when fermentations were ended. Yeast dry mass was determined from centrifuged and twice washed samples that were dried overnight at 105°C.

### Chemical Analysis of Wort and Beer

Concentrations of fermentable sugars (maltose and maltotriose) were measured by HPLC using a Waters 2695 Separation Module and Waters System Interphase Module liquid chromatograph coupled with a Waters 2414 differential refractometer (Waters Co., Milford, MA, United States). An Aminex HPX-87H organic acid analysis column (300 × 7.8 mm, Bio-Rad) was equilibrated with 5 mM H_2_SO_4_ (Titrisol, Merck, Germany) in water at 55°C and samples were eluted with 5 mM H_2_SO_4_ in water at a 0.3 mL/min flow rate.

The alcohol level (% v/v) of samples was determined from the centrifuged and degassed fermentation samples using an Anton Paar Density Meter DMA 5000 M with an Alcolyzer Beer ME module (Anton Paar GmbH, Austria).

Yeast-derived higher alcohols and esters were determined by headspace gas chromatography with flame ionization detector (HS-GC-FID) analysis. 4 mL samples were filtered (0.45 μm), incubated at 60°C for 30 min and then 1 mL of gas phase was injected (split mode; 225°C; split flow of 30 mL min^-1^) into a gas chromatograph equipped with an FID detector and headspace autosampler (Agilent 7890 Series; Palo Alto, CA, United States). Analytes were separated on a HP-5 capillary column (50 m × 320 μm × 1.05 μm column, Agilent, United States). The carrier gas was helium (constant flow of 1.4 mL min^-1^). The temperature program was 50°C for 3 min, 10°C min^-1^–100°C, 5°C min^-1^–140°C, 15°C min^-1^–260°C and then isothermal for 1 min. Compounds were identified by comparison with authentic standards and were quantified using standard curves. 1-Butanol was used as internal standard.

### Flocculation Assay

Flocculation of the yeast strains was evaluated using a modified Helm’s assay essentially as described by D’Hautcourt and Smart (1999). Yeast strains were grown overnight in 50 mL YP-Glucose at 24°C. The yeast was washed twice with 0.5 M EDTA (pH 7) to break the cell aggregates and then diluted to an OD600 of 0.4. Flocculation was assayed by first washing yeast pellets with 4 mM CaCl_2_ solution and resuspending them in 1 mL of flocculation solution containing 4 mM CaCl_2_, 6.8 g/L sodium acetate, 4.05 g/L acetic acid, and 4% (v/v) ethanol (pH 4.5). Yeast cells in control tubes were resuspended in 0.5 M EDTA (pH 7). After a sedimentation period of 10 min, samples (200 μL) were taken from just below the meniscus and dispersed in 10 mM EDTA (800 μL). The absorbance at 600 nm was measured, and percentage of flocculation was determined from the difference in absorbance between control and flocculation tubes. The assay was performed in triplicate.

### Melibiase Activity of Yeast

Melibiase activity was tested based on the ability to form blue-colored colonies when grown in the presence of X-α-Gal (#16555 Sigma-Aldrich) (ASBC, 2011).

### Dextrin Fermentation

The ability to ferment dextrin was tested in minimal growth media with dextrin as the sole carbon source. Strains were grown overnight in YP-Glucose, after which 2 mL microcentrifuge tubes containing 1 mL of dextrin media (0.67% YNB without amino acids, 1% dextrin from potato starch) were inoculated with 20 μL of the overnight cultures. The tubes were incubated at room temperature for 3 weeks, after which the refractive index of the culture media was measured with a Quick-Brix 90 digital refractometer (Mettler-Toledo AG, Switzerland). A decrease in refractive index indicated fermentation of dextrin. *S. cerevisiae* WLP590 (White Labs Inc, United States) and *S. pastorianus* VTT-A63015 (VTT culture collection, Finland) were included as positive and negative control strains, respectively. No change in refractive index was observed for the negative control strain. In addition, the presence of the *STA1* gene was tested with PCR using primers SD-5A and SD-6B (Yamauchi et al., 1998).

### Phenolic Off-Flavor Assay

The ability to produce POF was tested using the absorbance-based method described in Mertens et al. (2017). The test was performed in 2 mL microcentrifuge tubes containing 1 mL of media instead of 96-well plates as described in Mertens et al. (2017).

### Multiplex PCR With Species-Specific Primers

Amplification of the *S. cerevisiae*-specific *MEX67* gene (amplicon size 150 bp), *S. eubayanus*-specific *FSY1* gene (amplicon size 228 bp) and *S. uvarum*-specific *DBP6* gene (amplicon size 275 bp) was performed with PCR using the ScerF2, ScerR2, SeubF3, SeubR2, SbayF1, and SbayR1 primers described by Muir et al. (2011) and Pengelly and Wheals (2013).

### PCR-RFLP of COX2 to Determine Origin of mtDNA in Hybrids

Amplification of the mitochondrial *COX2* gene was performed with PCR using the COII-3 and COII-5 primers described by Belloch et al. (2000). The amplicon size (656 bp) of both the *S. cerevisiae*- and *S. uvarum*-derived *COX2* were of equal size, and they could therefore not be differentiated based on size. Digestion with the *HaeIII* restriction enzyme (New England Biolabs, United States) did not affect the *S. cerevisiae*-derived *COX2* amplicon, but yielded a 75 bp smaller fragment (581 bp) for the *S. uvarum*-derived *COX2* amplicon.

### Genome Sequencing and Analysis

The “Muri” strain was sequenced by Biomedicum Genomics (Helsinki, Finland). In brief, DNA was initially isolated using Qiagen 100/G Genomic tips (Qiagen, Netherlands), after which an Illumina TruSeq LT paired-end 150 bp library was prepared for each strain and sequencing was carried out with a NextSeq500 instrument. Paired-end reads from the NextSeq500 sequencing were quality-analyzed with FastQC (Andrews, 2010) and trimmed and filtered with Cutadapt (Martin, 2011). Reads were aligned to a concatenated reference genome of *S. cerevisiae* S288C (R64-2-1), *S. eubayanus* FM1318 (Baker et al., 2015) and *S. uvarum* CBS7001 (Scannell et al., 2011) using SpeedSeq (Chiang et al., 2015). Quality of alignments was assessed with QualiMap (García-Alcalde et al., 2012). Variant analysis was performed on aligned reads using FreeBayes (Garrison and Marth, 2012). Prior to variant analysis, alignments were filtered to a minimum MAPQ of 50 with SAMtools (Li et al., 2009). Variants at sites where read depth was below 10 were also excluded. Interchromosomal translocations were detected based on split reads with Manta (Chen et al., 2016), and visualized with the “circlize” package in R (Gu et al., 2014). The median coverage over 10,000 bp windows was calculated with BEDTools (Quinlan and Hall, 2010) and visualized in R. Gene ontology enrichment was performed with YeastMine (Balakrishnan et al., 2012). The raw sequencing reads are available in the NCBI’s Short Read Archive under BioProject PRJNA475668 in the NCBI BioProject database^1^.

### Phylogenetic Analysis

Prior to phylogenetic analysis, consensus genotypes of the *S. cerevisiae* and *S. uvarum* sub-genomes of the Muri strain were called from the identified variants using BCFtools (Li, 2011). Regions where the sequencing coverage was below 10 were excluded from the consensus genotypes. Genome assemblies of the 157 *S. cerevisiae* strains described in Gallone et al. (Gallone et al., 2016) were retrieved from NCBI (BioProject PRJNA323691). Consensus genotypes of 61 *S. uvarum* and hybrid strains described in Almeida et al. (2014) were kindly provided by José Paulo Sampaio. Multiple sequence alignment of the consensus genotype of the *S. cerevisiae* sub-genome of Muri and the 157 *S. cerevisiae* assemblies was performed with the NASP pipeline (Roe et al., 2016) using *S. cerevisiae* S288C (R64-2-1) as the reference genome. A matrix of single nucleotide polymorphisms (SNP) in the 159 strains was extracted from the aligned sequences. The SNPs were annotated with SnpEff (Cingolani et al., 2012) and filtered as follows: only sites that were in the coding sequence of genes, present in all 159 strains and with a minor allele frequency greater than 1% (one strain) were retained. The filtered matrix contained 3753194 SNPs (129434 sites). A maximum likelihood phylogenetic tree was estimated using IQ-TREE (Nguyen et al., 2015). IQ-TREE was run using the “GTR + F + R4” model and 1000 ultrafast bootstrap replicates (Minh et al., 2013). The resulting maximum likelihood tree was visualized in FigTree and rooted with *S. paradoxus* CBS432 (Yue et al., 2017). The above steps from multiple sequence alignment onward were repeated with the consensus genotypes of the *S. uvarum* strains (Almeida et al., 2014) and the *S. uvarum* sub-genome of Muri using *S. uvarum* CBS7001 as the reference genome (Scannell et al., 2011). The filtered matrix contained 2189200 SNPs (352638 sites).

### Data Visualization and Analysis

Data and statistical analyses were performed with R^2^. Z-scores (z) of the phenotypic traits were calculated as z = (x-μ)/σ, where x is value of a trait for a particular strain, μ is the mean value of that trait in all strains, and σ is the standard deviation of that trait in all strains. Heat maps with hierarchical clustering and optimal leaf ordering of the strains were generated based on the z-scores with the “seriation” package (Hahsler et al., 2008). Principal component analysis (PCA) was also performed on the set of z-scores. Prior to PCA, the z-scores from the concentrations of aroma compounds were scaled based on their flavor threshold as μ/C_threshold_, where μ is the mean concentration of that compound in all strains, and C_threshold_ is the aroma threshold of that compound (Meilgaard, 1982). This weighting was performed so that aroma compounds with concentrations much below the aroma threshold would have less impact on the PCA. Flow cytometry data was analyzed with “flowCore” (Hahne et al., 2009) and “mixtools” (Benaglia et al., 2009) packages. Plots were produced in R and FigTree.

### Data Availability

The Illumina reads generated in this study have been submitted to NCBI-SRA under BioProject number PRJNA475668 in the NCBI BioProject database (see footnote 1).

## Results

### Analysis of the Muri Genome

**FIGURE 1:**
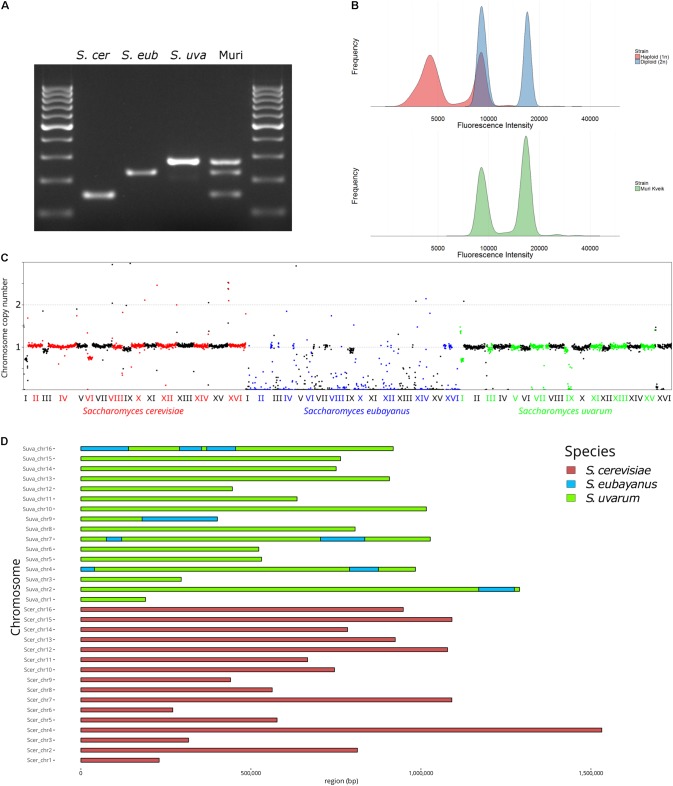
Characterization of the Muri strain **(A)** PCR with species-specific primers. *S. cer*: *S. cerevisiae*; *S. eub*: *S. eubayanus*; and *S. uva*: *S. uvarum*. **(B)** Fluorescence intensity of SYTOX Green-stained haploid and diploid control strains and the Muri hybrid during flow cytometry. **(C)** Normalized sequencing coverage of reads aligned to a concatenated *S. cerevisiae*, *S. eubayanus*, and *S. uvarum* reference genome. **(D)** A map of the 32 chromosomes in the Muri hybrid based on alignment to the reference genomes.

The genetic and phenotypic diversity of a range of Norwegian farmhouse brewing strains, i.e., kveik strains, was recently explored by Preiss et al. (2018). Sequencing of the ITS region identified the vast majority of the isolates as *S. cerevisiae*. However, one isolate, i.e., the Muri strain that is investigated in this study, was identified as a *S. bayanus*-type hybrid. To further characterize the genetic background of the Muri strain, we initially tested the species-specific multiplex PCR primer set described by Muir et al. (2011) and Pengelly and Wheals (2013). These primers yielded bands for *S. cerevisiae*, *S. eubayanus*, and *S. uvarum* (**Figure 1A**). Flow cytometry analysis further revealed that the Muri strain is approximately diploid (**Figure 1B**). We then sequenced the genome of the Muri strain with 150 bp paired-end Illumina sequencing. Based on the results from the species-specific primers, we first aligned the trimmed sequencing reads to a concatenated reference genome of *S. cerevisiae* S288C, *S. eubayanus* FM1318, and *S. uvarum* CBS7001 (**Figure 1C**). The alignment (263 × coverage and 97.3% mapped reads) suggested that the Muri strain is an allodiploid *S. cerevisiae* × *S. uvarum* hybrid, containing introgressions from *S. eubayanus* (**Supplementary Data S2**). Using Manta, we identified that the majority of these *S. eubayanus* introgressions were in the *S. uvarum* sub-genome (**Figure 1D** and **Supplementary Figure S1**). Interestingly, *S. uvarum* chromosome 9 appears to be a chimeric chromosome, where the right arm has been replaced by that of *S. eubayanus* chromosome 9 (the breakpoints identified by Manta are at positions 196846 and 180023 bp in the *S. uvarum* and *S. eubayanus* sequences, respectively). In addition, there were substantial contributions from *S. eubayanus* chromosomes 2, 4, 7, and 16 (**Figure 1D** and **Supplementary Data S2**). The mitochondria appeared to be of *S. uvarum* origin based on sequencing coverage when reads were aligned to the mitochondrial DNA (mtDNA) of *S. cerevisiae*, *S. eubayanus*, and *S. uvarum* (**Supplementary Table S1** and **Supplementary Figure S4C**).

**FIGURE 2:**
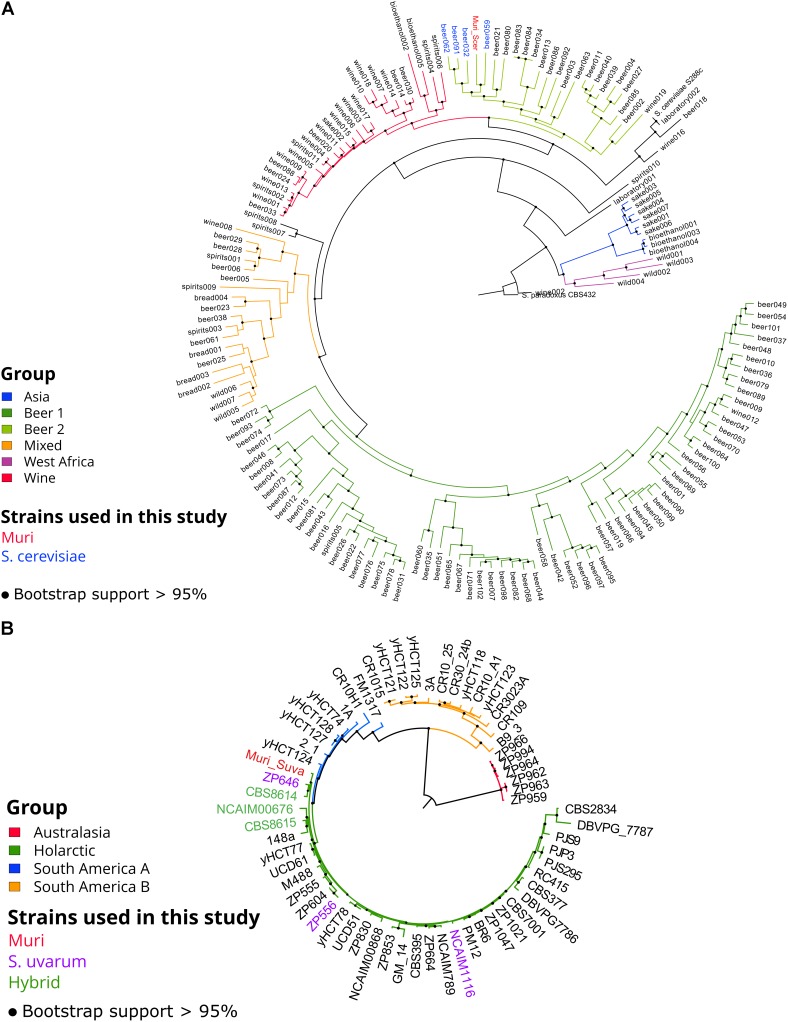
Phylogeny of Muri’s sub-genomes and the 157 *S. cerevisiae* strains described in Gallone et al. (2016) and 61 *S. uvarum* and hybrid strains described in Almeida et al. (2014). **(A)** Maximum likelihood phylogenetic tree based on SNPs at 129434 sites in 158 *S. cerevisiae* genomes (rooted with *S. paradoxus* as outgroup). **(B)** Maximum likelihood phylogenetic tree based on SNPs at 352638 sites in 62 *S. uvarum* and hybrid genomes (rooted with the Australasian strains as outgroup). Black dots on nodes indicate bootstrap support values >95%. Branches are colored according to lineage, and the names of strains used in this study (see **Table 1**) are colored (Muri, red; *S. cerevisiae*, blue; *S. uvarum*, purple; and Hybrids, green). Branch lengths represent the number of substitutions per site. Root branch lengths have been artificially shortened to improve the clarity of the trees.

Single nucleotide polymorphism (SNP) analysis of the aligned sequencing reads of the Muri strain revealed 48983 and 26439 SNPs in the haploid *S. cerevisiae* and *S. uvarum* sub-genomes, respectively, compared to the reference genomes. Allele frequency distributions of the SNPs suggest a single allele at each site, supporting the flow cytometry results of two haploid sub-genomes (**Supplementary Figure S2**). Consensus sequences of both sub-genomes were then produced from these SNPs (regions where coverage was below 10× were excluded). In order to identify *S. cerevisiae* and *S. uvarum* strains closely related to the parent strains of the Muri hybrid, we performed multiple sequence alignment, SNP identification and phylogenetic analysis using the NASP pipeline and IQ-TREE with the consensus sequences and 157 *S. cerevisiae* genome assemblies obtained from Gallone et al. (2016; Bioproject PRJNA323691), as well as 61 *S. uvarum* consensus sequences obtained from Almeida et al. (2014; Bioproject PRJNA230139). The inferred maximum-likelihood tree of the *S. cerevisiae* genomes, based on 129434 polymorphic sites, suggests that the *S. cerevisiae* sub-genome of the Muri strain belongs to a lineage of beer yeasts [“Beer 2” from Gallone et al. (2016) or “Mosaic Beer” from Peter et al. (2018)], with its closest relatives being ale yeasts (e.g., Beer059 and Beer032) from the United Kingdom (**Figure 2A**). These ale yeasts are characterized by good ethanol tolerance and production, as well as high flocculation (Gallone et al., 2016). In addition, many of these strains carry the *STA1* gene, encoding an extracellular glucoamylase that can cause super-attenuation during beer fermentation (Yamashita et al., 1985), as was revealed by a BLAST search of the genome assemblies (data not shown) and using PCR (**Supplementary Figure S3A**). The *STA1* gene was also identified in the Muri strain (**Supplementary Figure S3**). Clustering within the “Beer 2” yeasts, which have an estimated common ancestor at the end of the 17^th^ century (Gallone et al., 2016), would suggest a recent hybridization event for Muri. Likewise, the inferred maximum likelihood tree of the *S. uvarum* genomes, based on 352638 polymorphic sites, suggests the *S. uvarum* sub-genome of the Muri strain belongs to the Holarctic lineage (Almeida et al., 2014), with its closest relatives being domesticated Central European strains used in cider and wine fermentation (**Figure 2B**). Interestingly, the *S. uvarum* sub-genome of the Muri hybrid was closely related to the *S. uvarum* sub-genome of the CBS8614 triple hybrid (*S. cerevisiae* × *S. kudriavzevii* × *S. uvarum*). This triple hybrid was isolated from homemade apple cider produced in Brittany, France (Masneuf et al., 1998; Groth et al., 1999).

### Phenotypic Analysis of Muri and Closely Related Strains

**FIGURE 3:**
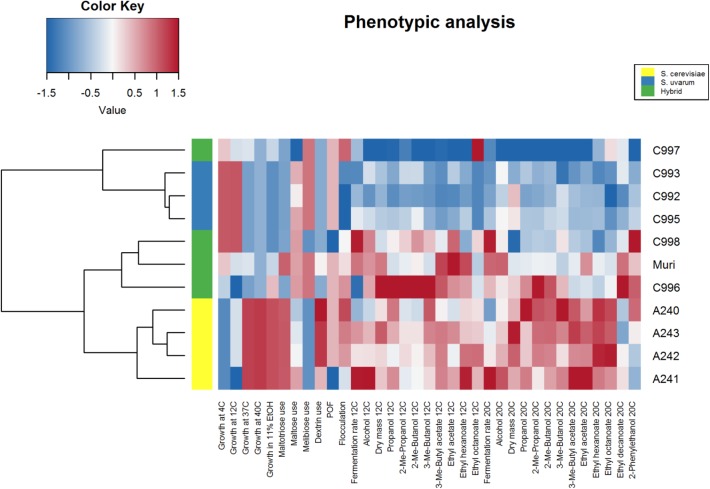
Heat map of 35 brewing-relevant phenotypic traits in *S. cerevisiae*, *S. uvarum*, and natural hybrid strains. The heat map was generated based on the z-scores (blue and red indicate low and high values, respectively). Strains were clustered using hierarchical clustering with optimal leaf ordering.

**FIGURE 4:**
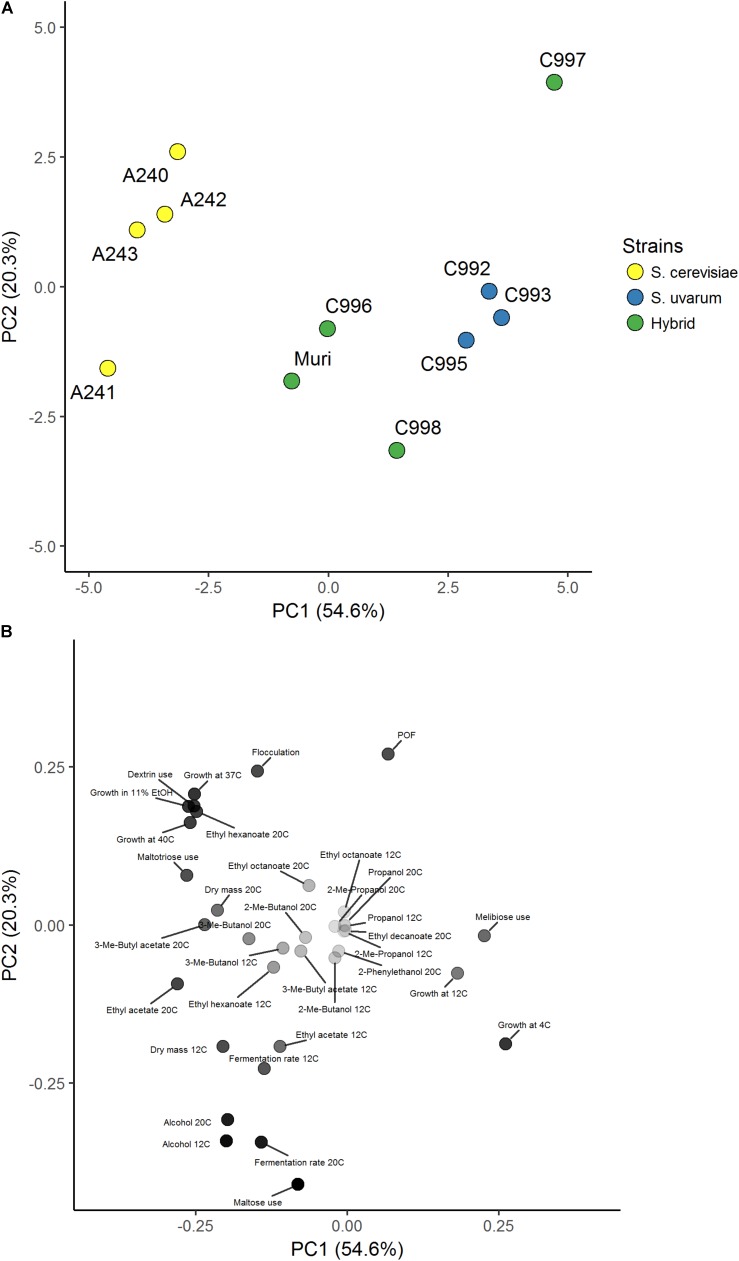
Principal component analysis of 35 brewing-relevant phenotypic traits in *S. cerevisiae*, *S. uvarum*, and natural hybrid strains. **(A)** Score plot of the 11 yeast strains in the first two principal components explaining 75% of the variance. Strains are colored based on species (*S. cerevisiae*: yellow, *S. uvarum*: blue, and hybrid: green). **(B)** Loadings plot of the 35 phenotypic traits. The aroma compounds were weighted based on the weighting factors in **Supplementary Tables S4, S5**. Points are colored based on distance to origin from white (zero distance) to black.

Four *S. cerevisiae* strains, three *S. uvarum* strains, and three interspecific hybrids that were genetically closely related to the Muri hybrid were obtained (**Table 1**). A comparative phenotypic analysis was performed on these eleven strains. Thirty-five traits were analyzed, including growth at various temperatures and on various carbon sources, fermentation performance in wort, and formation of aroma-active compounds. Hierarchical clustering with optimal leaf ordering based on the z-scores observed for the 35 traits grouped the 11 strains into three groups: one with the *S. cerevisiae* strains, one with the majority of the hybrid strains, and one with the *S. uvarum* strains and hybrid C997 (**Figure 3**). PCA on the same dataset also clustered the strains into the same three groups, with the exception that hybrid C997 was a clear outlier (**Figure 4**). The *S. cerevisiae* strains were separated from the *S. uvarum* strains along the first principal component, explaining 55% of the variance, while hybrids, with the exception of C997, grouped between them.

As was expected based on their brewing origin, the *S. cerevisiae* strains were associated with high flocculation, good fermentation performance in wort, high production of aroma compounds, and good growth at high temperatures and in 11% ethanol. The *S. uvarum* strains on the other hand were associated with good growth at lower temperatures and with melibiose as the sole carbon source. The hybrid strains tended to exhibit intermediate scores for the traits. Interestingly, the *S. cerevisiae* and *S. uvarum* strains that appeared most similar to the Muri hybrid based on the PCA (i.e., the shortest Euclidean distance to Muri in the first two principal components), A241 (Beer059) and C995 (ZP646), respectively, were also the strains that phylogenetically clustered closest to Muri’s sub-genomes (**Figure 2**).

### Recreating the Muri Strain Through Interspecific Hybridization

In an attempt to recreate the Muri hybrid, *de novo* interspecific yeast hybrids between *S. cerevisiae* A241 and *S. uvarum* C995 were generated by mating. Spore-to-spore mating was chosen over rare mating as the hybridization approach, since the resulting hybrids tend to be allodiploid. High sporulation efficiency was obtained for both parent strains, and a total of 12 confirmed hybrids were obtained from 60 attempted crosses. Hybrid confirmation was performed on single cell isolates (obtained from re-streaking colonies three times) using ITS-PCR with *HaeIII* digestion and species-specific primers (**Supplementary Figure S4**). The four fastest growing hybrids were selected for further analysis (listed in **Table 1**).

**FIGURE 5:**
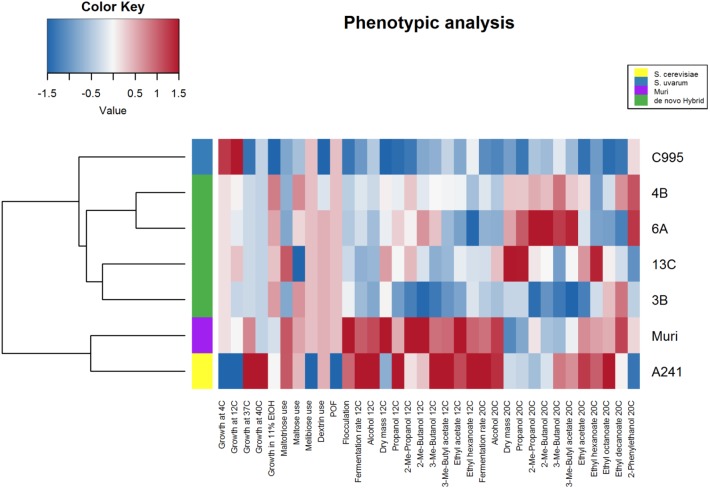
Heat map of 34 brewing-relevant phenotypic traits in Muri, *de novo* hybrid and parent strains. The heat map was generated based on the z-scores (blue and red indicate low and high values, respectively). Strains were clustered using hierarchical clustering with optimal leaf ordering.

**FIGURE 6:**
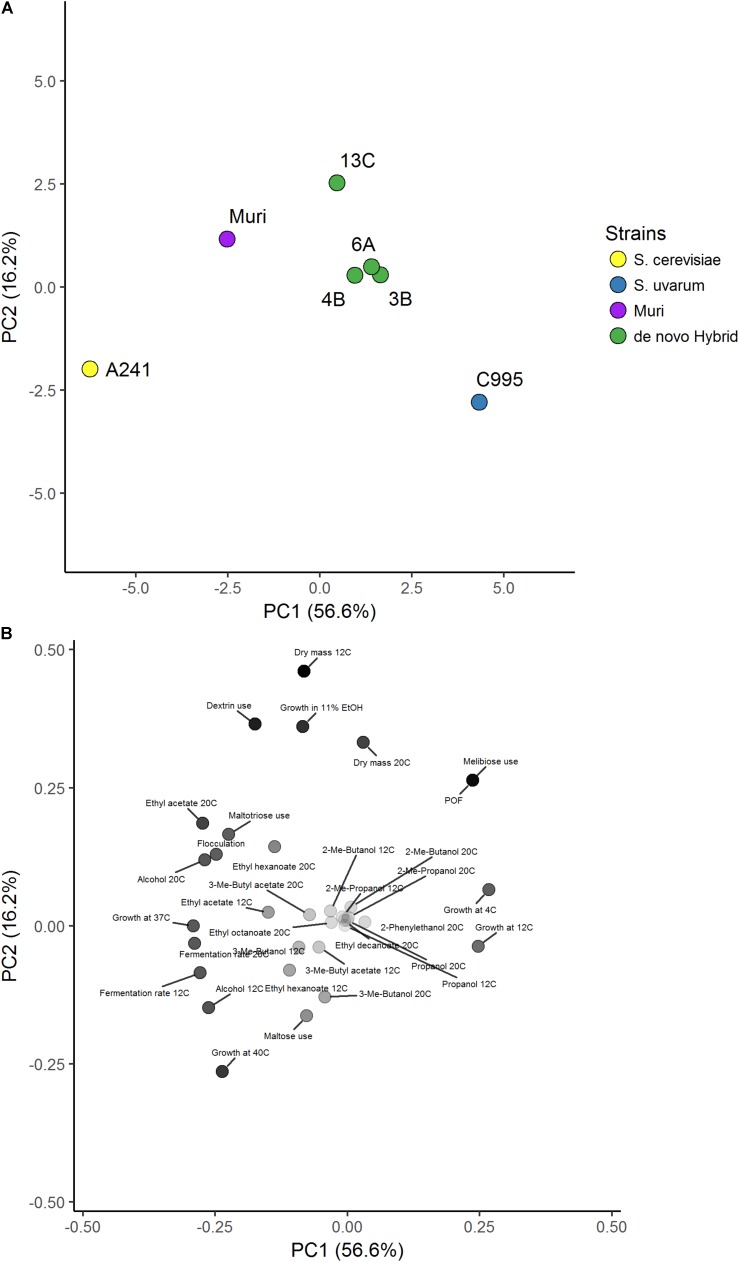
Principal component analysis of 34 brewing-relevant phenotypic traits in Muri, *de novo* hybrid and parent strains. **(A)** Score plot of the 7 yeast strains in the first two principal components explaining 73% of the variance. Strains are colored based on species (*S. cerevisiae*: yellow, *S. uvarum*: blue, Muri: purple, and *de novo* hybrid: green). **(B)** Loadings plot of the 34 phenotypic traits. The aroma compounds were weighted based on the weighting factors in **Supplementary Tables S6, S7**. Points are colored based on distance to origin from white (zero distance) to black.

In order to test how phenotypically similar the *de novo* hybrids were to the Muri hybrid, the comparative phenotypic analysis performed above was repeated with the four *de novo* hybrids, their parent strains, and the Muri hybrid. Hierarchical clustering with optimal leaf ordering based on the z-scores observed for 34 traits (ethyl octanoate concentrations from fermentations at 12°C were excluded because these compounds weren’t detected for multiple strains) grouped the *de novo* hybrids close to the *S. uvarum* C995 parent, while Muri was grouped with the *S. cerevisiae* A241 parent (**Figure 5**). PCA on the same dataset revealed that the *de novo* hybrids grouped with the Muri hybrid (**Figure 6**). The two parent strains were separated from each other along the first principal component, explaining 57% of the variance, while both Muri and the *de novo* hybrids grouped between them. The hybrids were separated from the parent strains along the second principal component, explaining 16% of the variance. The hybrid strains tended to show intermediate scores in the majority of the traits, while best-parent heterosis was observed for biomass formation and ability to grow in the presence of 11% ethanol. The *de novo* hybrids had also inherited the *STA1* gene and the ability to ferment dextrin from *S. cerevisiae* A241 (**Supplementary Figure S3B**). Across the studied set of phenotypic traits, the *de novo* hybrids 4B and 13C were more similar to Muri than either of the parent strains (**Supplementary Table S2**).

While the *de novo* hybrids performed similarly to Muri, there were differences in their performances, particularly during the low-temperature wort fermentations (**Figure 5**). Interestingly, despite growing well at 4 and 12°C, the *de novo* hybrids fermented wort slowly at 12°C. Furthermore, slight variation between the four *de novo* hybrids was also observed. Hybrid 13C, for example, was the only of the *de novo* hybrids which used maltotriose as efficiently as the A241 parent strain (**Figure 5**). We detected 23953 and 1490 heterozygous variants in the *S. cerevisiae* A241 (sequencing reads obtained from NCBI-SRA SRR5688171) and *S. uvarum* C995 (sequencing reads obtained from NCBI-SRA SRR1119163) parent strains, respectively. Of these, 10033 and 980, respectively, were detected in Muri as well. Therefore, one would expect spore siblings, and any resulting hybrids, to vary genetically and phenotypically. In addition, some diversity between the Muri hybrid and the *de novo* hybrids is also expected based on the sequence divergence between Muri’s sub-genomes and the parent strains A241 and C995. In the *S. cerevisiae* sub-genome of Muri, a total of 2623 missense and nonsense mutations were identified that were not present in A241, while A241 contained 2128 missense and nonsense mutations not present in Muri. Gene ontology enrichment of the list of genes that these mutations affected revealed that, compared to A241, Muri appeared to be affected by mutations in genes related to regulation (**Supplementary Table S3**). Similarly, 3413 unique missense and nonsense mutations that were not present in C995 were identified in the *S. uvarum* sub-genome of Muri. The inheritance of mtDNA also varied between the four *de novo* hybrids (**Supplementary Figure S4C**). Hybrid 6A had inherited mtDNA from the *S. uvarum* parent strain, while the other three strains had inherited mtDNA from the *S. cerevisiae* parent strain.

## Discussion

While natural *S. cerevisiae* × *S. eubayanus* and *S. cerevisiae* × *S. kudriavzevii* interspecies hybrids are frequently used in beer fermentations (González et al., 2008; Gibson and Liti, 2015), limited reports exist describing the use of *S. cerevisiae* × *S. uvarum* hybrids in brewing. Here we characterize and attempt to reconstruct a unique *S. cerevisiae* × *S. uvarum* natural hybrid, Muri, that was isolated from a Norwegian farmhouse brewing culture. Whole genome sequencing and phylogenetic analysis revealed that the Muri hybrid’s sub-genomes appeared to be closely related to domesticated *S. cerevisiae* and *S. uvarum* strains of British and Central European origin isolated from beer, cider and wine. Since the yeast was reportedly revived from an old yeast stock at the farmhouse (Garshol, 2013), we cannot exclude the possibility that the hybrid or one of its parents is a wild or contaminant yeast. However, the occurrence of wild *Saccharomyces* yeasts in Norway remains unexplored and no such strains are available for comparison. Interestingly, the *S. cerevisiae* sub-genome of the Muri strain does not appear to be related to other “kveik” isolates, which appear to be of “Beer 1” lineage rather than “Beer 2” (Preiss et al., 2018). It is therefore possible that the hybridization event to form Muri has occurred elsewhere, rather than at the farmhouse, or that not all kveik yeasts share the same ancestry. The allodiploid nature of Muri and the lack of structural rearrangements between the *S. cerevisiae* and the non-*S. cerevisiae* sub-genomes, are in contrast to that of other industrially used interspecific hybrids such as lager yeasts, which have been shown to exhibit considerable chromosomal copy number variations and rearrangements (van den Broek et al., 2015). This, together with “Beer 2” lineage of the *S. cerevisiae* sub-genome, suggests a more recent hybridization event in Muri compared to lager yeasts.

The Muri hybrid exhibited a range of phenotypic properties desirable for brewing. These included tolerance to both low and high temperatures, tolerance to a high ethanol concentration, efficient use of maltotriose, and formation of desirable aroma-active esters. In addition, the Muri hybrid possessed the *STA1* gene, encoding an extracellular glucoamylase enzyme (Yamashita et al., 1985), allowing it to utilize dextrin. This is a fairly unique property in brewing yeast and generally linked with beer spoilage (Meier-Dörnberg et al., 2018). However, it does allow for higher ethanol yield and the production of low-carbohydrate beer. The phenotype of Muri also makes it a potential candidate for other industrial fermentations, such as biofuel production or distilling, where good stress tolerance and high ethanol yield are desirable (Steensels et al., 2014b). Muri is not, however, a suitable candidate for lager beer production, as it possesses functional *PAD1* and *FDC1* genes. This allows it to produce POF, which are undesirable in lager beer. Interestingly, *S. cerevisiae* A241 (Beer059) did not produce POF as a result of premature stop codons in both *PAD1* and *FDC1* (Gallone et al., 2016). It would therefore be possible to construct POF-negative *de novo* hybrids with rare mating and sporulation using a fertile allotetraploid intermediate, as has been demonstrated with *S. cerevisiae* × *S. eubayanus* hybrids (Krogerus et al., 2017).

In an attempt to reconstruct the Muri hybrid, we generated *de novo* hybrids between *S. cerevisiae* A241 and *S. uvarum* C995. As was expected based on previous research (Bellon et al., 2011; Dunn et al., 2013; Steensels et al., 2014a; Krogerus et al., 2015; Mertens et al., 2015; Snoek et al., 2015), these hybrids inherited traits from both parent strains. These hybrids also appeared to successfully replicate the phenotype of Muri, with the exception of efficient wort fermentation at 12°C. It is likely that this is a result of impaired maltose transport, and the absence of cold-tolerant maltose permeases (Vidgren et al., 2010). This variation could also result from heterozygosity in the parent strains and sequence divergence relative to Muri’s sub-genomes. In addition, the origin of mtDNA in the *de novo* hybrids could influence their fitness (Wolters et al., 2018). The mtDNA in Muri appears to be from *S. uvarum*, while the majority of the *de novo* hybrids had inherited mtDNA from the *S. cerevisiae* parent. However, no obvious associations between the mitotype and phenotype were observed among the *de novo* hybrids. Recent studies with laboratory-generated *S. cerevisiae* × *S. uvarum* hybrids have revealed that transmission of mtDNA tends to be uniparental and a *S. cerevisiae* origin appears more common (Origone et al., 2018; Verspohl et al., 2018). However, mtDNA transmission appears strain-dependent.

A further potential cause of deviation between Muri and the *de novo* hybrids, is the impact of *S. eubayanus* introgressions. These introgressions are common in Holarctic *S. uvarum* strains, but the introgressed regions differ significantly depending on substrate origin and appear more common in domesticated strains (Almeida et al., 2014; Albertin et al., 2018). For example, an introgressed region (40 kb) from the left arm of *S. eubayanus* chromosome IV containing *FSY1*, a gene that enables efficient fructose transport, was detected in Muri, but not in *S. uvarum* C995 or any other *S. uvarum* strain studied by Almeida et al. (2014). The *S. eubayanus*-specific primers described by Pengelly and Wheals (2013) were designed based on the *S. eubayanus* allele of *FSY1*, which explains the PCR band for *S. eubayanus* that was detected in Muri (**Figure 1A**). The phenotypic impacts of *S. eubayanus* introgressions in *S. uvarum* have not yet been elucidated, but Albertin et al. (2018) speculate that they could be the most important source of genetic and phenotypic variability in Holarctic *S. uvarum* strains. These impacts should therefore be clarified in future studies. Furthermore, the use of long read sequencing technologies (e.g., PacBio or Nanopore) could also be applied to the Muri hybrid to generate end-to-end genome assemblies (Yue et al., 2017) in order to improve the detection of structural rearrangements and features not present in the reference genomes.

The approach used here, i.e., combining phylogenetic and phenotypic analysis to aid in reconstructing a natural hybrid, could be particularly useful if applied to lager yeast. While no single strain has yet been identified as the potential *S. cerevisiae* ale parent of lager yeast, recent whole genome studies suggest that the last common ancestor of the *S. cerevisiae* sub-genome of lager yeasts is found among the “Beer 1” or “Ale beer” yeasts, close to the German wheat beer strains (Gonçalves et al., 2016; Monerawela and Bond, 2018). Such *de novo* hybrids could be used in evolutionary engineering studies to investigate which environmental conditions cause genomic changes that mimic those that have occurred in natural lager yeast. Saaz-type lager yeast, for example, have retained a larger fraction of the *S. eubayanus* sub-genome than the *S. cerevisiae* sub-genome (Dunn and Sherlock, 2008; Okuno et al., 2015), and it is still unclear how the environment has impacted its evolution. Evolutionary engineering studies with *de novo* interspecific hybrids have shown that either of the parental sub-genomes may be preferentially retained during stabilization (Piotrowski et al., 2012; Dunn et al., 2013; Lopandic et al., 2016; Smukowski Heil et al., 2017; Krogerus et al., 2018), and a stressful environment may cause more drastic changes. Exposing *S. cerevisiae* × *S. uvarum* hybrids to high temperatures resulted in the loss of the heat-sensitive *S. uvarum* sub-genome (Piotrowski et al., 2012), while exposing *S. cerevisiae* × *S. eubayanus* hybrids to high ethanol concentrations resulted in a greater loss of the *S. eubayanus* sub-genome (Krogerus et al., 2018). Evolutionary engineering of *de novo* hybrids constructed from the last common ancestors of natural lager yeasts’ sub-genomes could therefore help elucidate how various environmental stresses and nutrient limitations have shaped their genomes.

## Conclusion

In conclusion, we show that the Muri hybrid that was isolated from a Norwegian farmhouse beer is an allodiploid *S. cerevisiae* × *S. uvarum* hybrid. Phylogenetic analysis of the sub-genomes of this hybrid indicated that its *S. cerevisiae* parent was of brewing origin. The Muri strain possesses a range of industrially desirable phenotypic properties, making it an interesting candidate for not only brewing applications, but potentially various other industrial fermentations, such as biofuel production and distilling. In addition, we show that it is possible to mimic the phenotype of this natural hybrid, by constructing *de novo* hybrids using parent strains closely related to Muri’s sub-genomes. This novel approach to studying natural hybrid strains has uses in both strain development and elucidating the evolutionary history of natural hybrids.

## Author Contributions

KK, BG, and RP designed the experiments. KK conducted the experiments described in this study, and analyzed all data. RP contributed the Muri hybrid. KK and BG wrote the manuscript. All authors read and approved the final manuscript.

## Conflict of Interest Statement

RP was employed by Escarpment Laboratories Inc., BG was employed by VTT Technical Research Centre of Finland Ltd., and KK is affiliated with VTT Technical Research Centre of Finland Ltd. All authors declare no competing interests. The funders had no role in study design, data collection and analysis, decision to publish, or preparation of the manuscript.

## Footnotes

1 https://www.ncbi.nlm.nih.gov/bioproject/

2 http://www.r-project.org/

## References

[B1] Albertin W. Chernova M. Durrens P. Guichoux E. Sherman D. J. Masneuf-Pomarede I. (2018). Many interspecific chromosomal introgressions are highly prevalent in Holarctic *Saccharomyces uvarum* strains found in human-related fermentations. *Yeast* 35 141–156. 10.1002/yea.3248 28779574

[B2] Alexander W. G. Peris D. Pfannenstiel B. T. Opulente D. A. Kuang M. Hittinger C. T. (2016). Efficient engineering of marker-free synthetic allotetraploids of Saccharomyces. *Fungal Genet. Biol.* 89 10–17. 10.1016/j.fgb.2015.11.002 26555931 PMC4789119

[B3] Almeida P. Gonçalves C. Teixeira S. Libkind D. Bontrager M. Masneuf-Pomarède I. (2014). A Gondwanan imprint on global diversity and domestication of wine and cider yeast *Saccharomyces uvarum*. *Nat. Commun.* 5:4044. 10.1038/ncomms5044 24887054 PMC5081218

[B4] Andrews S. (2010). *FastQC: A Quality Control Tool for High Throughput Sequence Data.* Available at: http://www.bioinformatics.babraham.ac.uk/projects/

[B5] Aquilani B. Laureti T. Poponi S. Secondi L. (2015). Beer choice and consumption determinants when craft beers are tasted: An exploratory study of consumer preferences. *Food Qual. Prefer.* 41 214–224. 10.1016/j.foodqual.2014.12.005

[B6] ASBC (2011). *ASBC Methods of Analysis.* St. Paul, MI: American Society of Brewing Chemists.

[B7] Baker E. Wang B. Bellora N. Peris D. Hulfachor A. B. Koshalek J. A. (2015). The genome sequence of *saccharomyces eubayanus* and the domestication of lager-brewing yeasts. *Mol. Biol. Evol.* 32 2818–2831. 10.1093/molbev/msv168 26269586 PMC4651232

[B8] Balakrishnan R. Park J. Karra K. Hitz B. C. Binkley G. Hong E. L. (2012). YeastMine-An integrated data warehouse for *Saccharomyces cerevisiae* data as a multipurpose tool-kit. *Database* 2012:bar062. 10.1093/database/bar062 22434830 PMC3308152

[B9] Belloch C. Querol A. García M. D. Barrio E. (2000). Phylogeny of the genus Kluyveromyces inferred from the mitochondrial cytochrome-c oxidase II gene. *Int. J. Syst. Evol. Microbiol.* 50 405–416. 10.1099/00207713-50-1-405 10826829

[B10] Bellon J. R. Eglinton J. M. Siebert T. E. Pollnitz A. P. Rose L. De Barros Lopes M. (2011). Newly generated interspecific wine yeast hybrids introduce flavour and aroma diversity to wines. *Appl. Microbiol. Biotechnol.* 91 603–612. 10.1007/s00253-011-3294-3 21538112

[B11] Benaglia T. Chauveau D. Hunter D. R. Young D. (2009). Mixtools: an R package for analyzing finite mixture models. *J. Stat. Softw.* 32 1–29. 10.18637/jss.v032.i06

[B12] Chen X. Schulz-Trieglaff O. Shaw R. Barnes B. Schlesinger F. Källberg M. (2016). Manta: rapid detection of structural variants and indels for germline and cancer sequencing applications. *Bioinformatics* 32 1220–1222. 10.1093/bioinformatics/btv710 26647377

[B13] Chiang C. Layer R. M. Faust G. G. Lindberg M. R. Rose D. B. Garrison E. P. (2015). SpeedSeq: ultra-fast personal genome analysis and interpretation. *Nat. Methods* 12 1–5. 10.1038/nmeth.3505 26258291 PMC4589466

[B14] Christine L. J. Marc L. Catherine D. Claude E. Jean-Luc L. Michel A. (2007). Characterization of natural hybrids of *Saccharomyces cerevisiae* and *Saccharomyces bayanus* var. *uvarum*. *FEMS Yeast Res.* 7 540–549. 10.1111/j.1567-1364.2007.00207.x 17302940

[B15] Cingolani P. Platts A. Wang L. L. Coon M. Nguyen T. Wang L. (2012). A program for annotating and predicting the effects of single nucleotide polymorphisms, SnpEff: SNPs in the genome of Drosophila melanogaster strain w1118; iso-2; iso-3. *Fly* 6 80–92. 10.4161/fly.19695 22728672 PMC3679285

[B16] D’Hautcourt O. Smart K. A. (1999). Measurement of brewing yeast flocculation. *J. Am. Soc. Brew. Chem.* 57 123–128. 10.1094/ASBCJ-57-0129

[B17] Dunn B. Paulish T. Stanbery A. Piotrowski J. Koniges G. Kroll E. (2013). Recurrent rearrangement during adaptive evolution in an interspecific yeast hybrid suggests a model for rapid introgression. *PLoS Genet.* 9:e1003366. 10.1371/journal.pgen.1003366 23555283 PMC3605161

[B18] Dunn B. Sherlock G. (2008). Reconstruction of the genome origins and evolution of the hybrid lager yeast *Saccharomyces pastorianus*. *Genome Res.* 18 1610–1623. 10.1101/gr.076075.108 18787083 PMC2556262

[B19] Fowle Z. (2017). *Kveik: The Hottest New Centuries-old Beer Yeast You’ve Never Heard of. Draft Mag.* Available at: http://draftmag.com/kveik-the-hottest-new-centuries-old-beer-yeast-youve-never-heard-of/

[B20] Gallone B. Steensels J. Baele G. Maere S. Verstrepen K. J. Prahl T. (2016). Domestication and divergence of *Saccharomyces cerevisiae* beer yeasts. *Cell* 166 1397 e.16–1410 e.16. 10.1016/j.cell.2016.08.020 27610566 PMC5018251

[B21] García-Alcalde F. Okonechnikov K. Carbonell J. Cruz L. M. Götz S. Tarazona S. (2012). Qualimap: evaluating next-generation sequencing alignment data. *Bioinformatics* 28 2678–2679. 10.1093/bioinformatics/bts503 22914218

[B22] Garrison E. Marth G. (2012). Haplotype-based variant detection from short-read sequencing. arXiv:1207.3907v2 [Preprint].

[B23] Garshol L. M. (2013). *Kveik: Norwegian Farmhouse Yeast.* Available at: http://www.garshol.priv.no/blog/264.html

[B24] Garshol L. M. (2016). *Gårdsøl - Det Norske Ølet.* Oslo: Cappelen Damm.

[B25] Gibson B. Geertman J.-M. A. Hittinger C. T. Krogerus K. Libkind D. Louis E. J. (2017). New yeasts—new brews: modern approaches to brewing yeast design and development. *FEMS Yeast Res.* 17:fox038. 10.1093/femsyr/fox038 28582493

[B26] Gibson B. Liti G. (2015). *Saccharomyces pastorianus*: Genomic insights inspiring innovation for industry. *Yeast* 32 17–27. 10.1002/yea.3033 25088523

[B27] Gonçalves M. Pontes A. Almeida P. Barbosa R. Serra M. Libkind D. (2016). Distinct domestication trajectories in top-fermenting beer yeasts and wine yeasts. *Curr. Biol.* 26 2750–2761. 10.1016/j.cub.2016.08.040 27720622

[B28] González S. S. Barrio E. Querol A. (2008). Molecular characterization of new natural hybrids of *Saccharomyces cerevisiae* and *S. kudriavzevii* in brewing. *Appl. Environ. Microbiol.* 74 2314–2320. 10.1128/AEM.01867-07 18296532 PMC2293171

[B29] Groth C. Hansen J. Piskur J. (1999). A natural chimeric yeast containing genetic material from three species. *Int. J. Syst. Bacteriol.* 49(Pt 4), 1933–1938. 10.1099/00207713-49-4-1933 10555378

[B30] Gu Z. Gu L. Eils R. Schlesner M. Brors B. (2014). Circlize implements and enhances circular visualization in R. *Bioinformatics* 30 2811–2812. 10.1093/bioinformatics/btu393 24930139

[B31] Hahne F. LeMeur N. Brinkman R. R. Ellis B. Haaland P. Sarkar D. (2009). flowCore: a Bioconductor package for high throughput flow cytometry. *BMC Bioinformatics* 10:106. 10.1186/1471-2105-10-106 19358741 PMC2684747

[B32] Hahsler M. Hornik K. Buchta C. (2008). Getting Things in Order?: An Introduction to the R Package seriation. *J. Stat. Softw.* 25 1–27. 10.1088/0031-9120/44/5/F04

[B33] Hebly M. Brickwedde A. Bolat I. Driessen M. R. M. de Hulster E. A. F. van den Broek M. (2015). S. *cerevisiae × S.* eubayanus interspecific hybrid, the best of both worlds and beyond. *FEMS Yeast Res.* 15 1–14. 10.1093/femsyr/fov005 25743788

[B34] Krogerus K. Arvas M. De Chiara M. Magalhães F. Mattinen L. Oja M. (2016). Ploidy influences the functional attributes of de novo lager yeast hybrids. *Appl. Microbiol. Biotechnol.* 100 7203–7222. 10.1007/s00253-016-7588-3 27183995 PMC4947488

[B35] Krogerus K. Holmström S. Gibson B. (2018). Enhanced wort fermentation with de novo lager hybrids adapted to high-ethanol environments. *Appl. Environ. Microbiol.* 84 e2302–e2317. 10.1128/AEM.02302-17 29196294 PMC5795086

[B36] Krogerus K. Magalhães F. Vidgren V. Gibson B. (2015). New lager yeast strains generated by interspecific hybridization. *J. Ind. Microbiol. Biotechnol.* 42 769–778. 10.1007/s10295-015-1597-6 25682107 PMC4412690

[B37] Krogerus K. Seppänen-Laakso T. Castillo S. Gibson B. (2017). Inheritance of brewing-relevant phenotypes in constructed *Saccharomyces cerevisiae* x *Saccharomyces eubayanus* hybrids. *Microb. Cell Fact.* 16:66. 10.1186/s12934-017-0679-8 28431563 PMC5399851

[B38] Li H. (2011). A statistical framework for SNP calling, mutation discovery, association mapping and population genetical parameter estimation from sequencing data. *Bioinformatics* 27 2987–2993. 10.1093/bioinformatics/btr509 21903627 PMC3198575

[B39] Li H. Handsaker B. Wysoker A. Fennell T. Ruan J. Homer N. (2009). The sequence alignment/map format and SAMtools. *Bioinformatics* 25 2078–2079. 10.1093/bioinformatics/btp352 19505943 PMC2723002

[B40] Lopandic K. (2018). Saccharomyces interspecies hybrids as model organisms for studying yeast adaptation to stressful environments. *Yeast* 35 21–38. 10.1002/yea.3294 29131388

[B41] Lopandic K. Pfliegler W. P. Tiefenbrunner W. Gangl H. Sipiczki M. Sterflinger K. (2016). Genotypic and phenotypic evolution of yeast interspecies hybrids during high-sugar fermentation. *Appl. Microbiol. Biotechnol.* 100 6331–6343. 10.1007/s00253-016-7481-0 27075738

[B42] Martin M. (2011). Cutadapt removes adapter sequences from high-throughput sequencing reads. *EMBnet.journal* 17:10 10.14806/ej.17.1.200

[B43] Masneuf I. Hansen J. Groth C. Piskur J. Dubourdieu D. (1998). New hybrids between *Saccharomyces* sensu stricto yeast species found among wine and cider production strains. *Appl. Environ. Microbiol.* 64 3887–3892. 9758815 10.1128/aem.64.10.3887-3892.1998 PMC106574

[B44] Meier-Dörnberg T. Kory O. I. Jacob F. Michel M. Hutzler M. (2018). *Saccharomyces cerevisiae* variety diastaticus friend or foe?—spoilage potential and brewing ability of different *Saccharomyces cerevisiae* variety diastaticus yeast isolates by genetic, phenotypic and physiological characterization. *FEMS Yeast Res* 18 foy023. 10.1093/femsyr/foy023 29518233

[B45] Meilgaard M. C. (1982). Prediction of flavor differences between beers from their chemical composition. *J. Agric. Food Chem.* 30 1009–1017. 10.1021/jf00114a002

[B46] Mertens S. Steensels J. Gallone B. Souffriau B. Malcorps P. Verstrepen K. J. (2017). Rapid Screening Method for Phenolic Off-Flavor (POF) Production in Yeast. *J. Am. Soc. Brew. Chem.* 75 318–323. 10.1094/ASBCJ-2017-4142-01

[B47] Mertens S. Steensels J. Saels V. De Rouck G. Aerts G. Verstrepen K. J. (2015). A large set of newly created interspecific Saccharomyces hybrids increases aromatic diversity in lager beers. *Appl. Environ. Microbiol.* 81 8202–8214. 10.1128/AEM.02464-15 26407881 PMC4651086

[B48] Minh B. Q. Nguyen M. A. T. Von Haeseler A. (2013). Ultrafast approximation for phylogenetic bootstrap. *Mol. Biol. Evol.* 30 1188–1195. 10.1093/molbev/mst024 23418397 PMC3670741

[B49] Monerawela C. Bond U. (2018). The hybrid genomes of *Saccharomyces pastorianus*: A current perspective. *Yeast* 35 39–50. 10.1002/yea.3250 28787090

[B50] Muir A. Harrison E. Wheals A. (2011). A multiplex set of species-specific primers for rapid identification of members of the genus Saccharomyces. *FEMS Yeast Res.* 11 552–563. 10.1111/j.1567-1364.2011.00745.x 22093682

[B51] Nguyen H. V. Legras J. L. Neuvéglise C. Gaillardin C. (2011). Deciphering the hybridisation history leading to the lager lineage based on the mosaic genomes of *Saccharomyces bayanus* strains NBRC1948 and CBS380 T. *PLoS ONE* 6:e25821. 10.1371/journal.pone.0025821 21998701 PMC3187814

[B52] Nguyen L. T. Schmidt H. A. Von Haeseler A. Minh B. Q. (2015). IQ-TREE: A fast and effective stochastic algorithm for estimating maximum-likelihood phylogenies. *Mol. Biol. Evol.* 32 268–274. 10.1093/molbev/msu300 25371430 PMC4271533

[B53] Nikulin J. Krogerus K. Gibson B. (2018). Alternative Saccharomyces interspecies hybrid combinations and their potential for low-temperature wort fermentation. *Yeast* 35 113–127. 10.1002/yea.3246 28755430 PMC5811906

[B54] Okuno M. Kajitani R. Ryusui R. Morimoto H. Kodama Y. Itoh T. (2015). Next-generation sequencing analysis of lager brewing yeast strains reveals the evolutionary history of interspecies hybridization. *DNA Res* 23 67–80. 10.1093/dnares/dsv037 26732986 PMC4755528

[B55] Origone A. C. Rodríguez M. E. Oteiza J. M. Querol A. Lopes C. A. (2018). *Saccharomyces cerevisiae* × *Saccharomyces uvarum* hybrids generated under different conditions share similar winemaking features. *Yeast* 35 157–171. 10.1002/yea.3295 29131448

[B56] Pengelly R. J. Wheals A. E. (2013). Rapid identification of *Saccharomyces eubayanus* and its hybrids. *FEMS Yeast Res.* 13 156–161. 10.1111/1567-1364.12018 23110474

[B57] Peris D. Moriarty R. V. Alexander W. G. Baker E. Sylvester K. Sardi M. (2017). Hybridization and adaptive evolution of diverse *Saccharomyces* species for cellulosic biofuel production. *Biotechnol. Biofuels* 10:78. 10.1186/s13068-017-0763-7 28360936 PMC5369230

[B58] Peter J. De Chiara M. Friedrich A. Yue J.-X. Pflieger D. Bergström A. (2018). Genome evolution across 1,011 *Saccharomyces cerevisiae* isolates. *Nature* 556 339–344. 10.1038/s41586-018-0030-5 29643504 PMC6784862

[B59] Pham T. Wimalasena T. Box W. G. Koivuranta K. Storgårds E. Smart K. A. (2011). Evaluation of ITS PCR and RFLP for differentiation and identification of brewing yeast and brewery “wild” yeast contaminants. *J. Inst. Brew.* 117 556–568. 10.1002/j.2050-0416.2011.tb00504.x PMC7197508 32834175

[B60] Piotrowski J. S. Nagarajan S. Kroll E. Stanbery A. Chiotti K. E. Kruckeberg A. L. (2012). Different selective pressures lead to different genomic outcomes as newly-formed hybrid yeasts evolve. *BMC Evol. Biol.* 12:46. 10.1186/1471-2148-12-46 22471618 PMC3372441

[B61] Plech M. de Visser J. A. G. M. Korona R. (2014). Heterosis is prevalent among domesticated but not wild strains of *Saccharomyces cerevisiae*. *G* 3 315–323. 10.1534/g3.113.009381 24347627 PMC3931565

[B62] Preiss R. Tyrawa C. Krogerus K. Garshol L. M. van der Merwe G. (2018). Traditional Norwegian kveik are a genetically distinct group of domesticated *Saccharomyces cerevisiae* brewing yeasts. *Front. Microbiol.* 9:2137 10.3389/fmicb.2018.02137 PMC6145013 30258422

[B63] Quinlan A. R. Hall I. M. (2010). BEDTools: A flexible suite of utilities for comparing genomic features. *Bioinformatics* 26 841–842. 10.1093/bioinformatics/btq033 20110278 PMC2832824

[B64] Rainieri S. Kodama Y. Kaneko Y. Mikata K. Nakao Y. Ashikari T. (2006). Pure and mixed genetic lines of *Saccharomyces bayanus* and *Saccharomyces pastorianus* and their contribution to the lager brewing strain genome. *Appl. Environ. Microbiol.* 72 3968–3974. 10.1128/AEM.02769-05 16751504 PMC1489639

[B65] Roe C. Smith D. E. Williamson C. H. D. Aziz M. Keim P. Hepp C. M. (2016). NASP: an accurate, rapid method for the identification of SNPs in WGS datasets that supports flexible input and output formats. *Microb. Genomics* 2:e000074. 10.1099/mgen.0.000074 28348869 PMC5320593

[B66] Sato M. Kishimoto M. Watari J. Takashio M. (2002). Breeding of brewer’s yeast by hybridization between a top-fermenting yeast *Saccharomyces cerevisiae* and a cryophilic yeast *Saccharomyces bayanus*. *J. Biosci. Bioeng.* 93 509–511. 10.1016/S1389-1723(02)80101-r3 16233241

[B67] Scannell D. R. Zill O. A. Rokas A. Payen C. Dunham M. J. Eisen M. B. (2011). The awesome power of yeast evolutionary genetics: new genome sequences and strain resources for the *Saccharomyces* sensu stricto Genus. *G*3 1 11–25. 10.1534/g3.111.000273 22384314 PMC3276118

[B68] Smukowski Heil C. S. DeSevo C. G. Pai D. A. Tucker C. M. Hoang M. L. Dunham M. J. (2017). Loss of heterozygosity drives adaptation in hybrid yeast. *Mol. Biol. Evol.* 34 1596–1612. 10.1093/molbev/msx098 28369610 PMC5455960

[B69] Snoek T. Picca Nicolino M. Van den Bremt S. Mertens S. Saels V. Verplaetse A. (2015). Large-scale robot-assisted genome shuffling yields industrial *Saccharomyces cerevisiae* yeasts with increased ethanol tolerance. *Biotechnol. Biofuels* 8:32. 10.1186/s13068-015-0216-0 25759747 PMC4354739

[B70] Steensels J. Meersman E. Snoek T. Saels V. Verstrepen K. J. (2014a). Large-scale selection and breeding to generate industrial yeasts with superior aroma production. *Appl. Environ. Microbiol.* 80 6965–6975. 10.1128/AEM.02235-14 25192996 PMC4249010

[B71] Steensels J. Snoek T. Meersman E. Nicolino M. P. Voordeckers K. Verstrepen K. J. (2014b). Improving industrial yeast strains: Exploiting natural and artificial diversity. *FEMS Microbiol. Rev.* 38 947–995. 10.1111/1574-6976.12073 24724938 PMC4293462

[B72] van den Broek M. Bolat I. Nijkamp J. F. Ramos E. Luttik M. A. H. Koopman F. (2015). Chromosomal copy number variation in *Saccharomyces pastorianus* is evidence for extensive genome dynamics in industrial lager brewing strains. *Appl. Environ. Microbiol.* 81 6253–6267. 10.1128/AEM.01263-15 26150454 PMC4542246

[B73] Verspohl A. Pignedoli S. Giudici P. (2018). The inheritance of mitochondrial DNA in interspecific Saccharomyces hybrids and their properties in winemaking. *Yeast* 35 173–187. 10.1002/yea.3288 29048749

[B74] Vidgren V. Multanen J. P. Ruohonen L. Londesborough J. (2010). The temperature dependence of maltose transport in ale and lager strains of brewer’s yeast. *FEMS Yeast Res.* 10 402–411. 10.1111/j.1567-1364.2010.00627.x 20402791 PMC2878602

[B75] Wolters J. F. Charron G. Gaspary A. Landry C. R. Fiumera A. C. Fiumera H. L. (2018). Mitochondrial recombination reveals mito–mito epistasis in yeast. *Genetics* 209 307–319. 10.1534/genetics.117.300660 29531011 PMC5937185

[B76] Yamashita I. Maemura T. Hatano T. Fukui S. (1985). Polymorphic extracellular glucoamylase genes and their evolutionary origin in the yeast Saccharomyces diastaticus. *J. Bacteriol.* 161 574–582. 3918018 10.1128/jb.161.2.574-582.1985 PMC214921

[B77] Yamauchi H. Yamamoto H. Shibano Y. Amaya N. Saeki T. (1998). Rapid methods for detecting Saccharomyces diastaticus, a beer spoilage yeast, using the polymerase chain reaction. *J. Am. Soc. Brew. Chem.* 56 58–63. 10.1094/ASBCJ-56-0058

[B78] Yue J. X. Li J. Aigrain L. Hallin J. Persson K. Oliver K. (2017). Contrasting evolutionary genome dynamics between domesticated and wild yeasts. *Nat. Genet.* 49 913–924. 10.1038/ng.3847 28416820 PMC5446901

