# Supplementary material for: A Unique Saccharomyces cerevisiae × Saccharomyces uvarum Hybrid Isolated From Norwegian Farmhouse Beer: Characterization and Reconstruction
Source: Front Microbiol. 2018 Sep 24;9:2253. doi: 10.3389/fmicb.2018.02253 (PMC6165869; doi:10.3389/fmicb.2018.02253)
Supplement: Supplementary file 2 [file Data_Sheet_1.pdf]

Supplementary Material

A unique Saccharomyces cerevisiae × Saccharomyces uvarum hybrid isolated from Norwegian farmhouse beer: characterization and reconstruction

Kristoffer Krogerus[1,2#], Richard Preiss[3,4], Brian Gibson[1]

[1] VTT Technical Research Centre of Finland, Tietotie 2, P.O. Box 1000, FI-02044 VTT, Espoo, Finland

[2] Department of Biotechnology and Chemical Technology, Aalto University, School of Chemical Technology, Kemistintie 1, Aalto, P.O. Box 16100, FI-00076 Espoo, Finland

[3] Department of Molecular and Cellular Biology, University of Guelph, 50 Stone Rd. E, Guelph, ON N1G 2W1, Canada

[4] Escarpment Laboratories, 8 Smith Ave., Guelph, ON N1E 5Y5, Canada

# Address correspondence to Kristoffer Krogerus, kristoffer.krogerus@gmail.com

DOI: 10.3389/fmicb.2018.02253

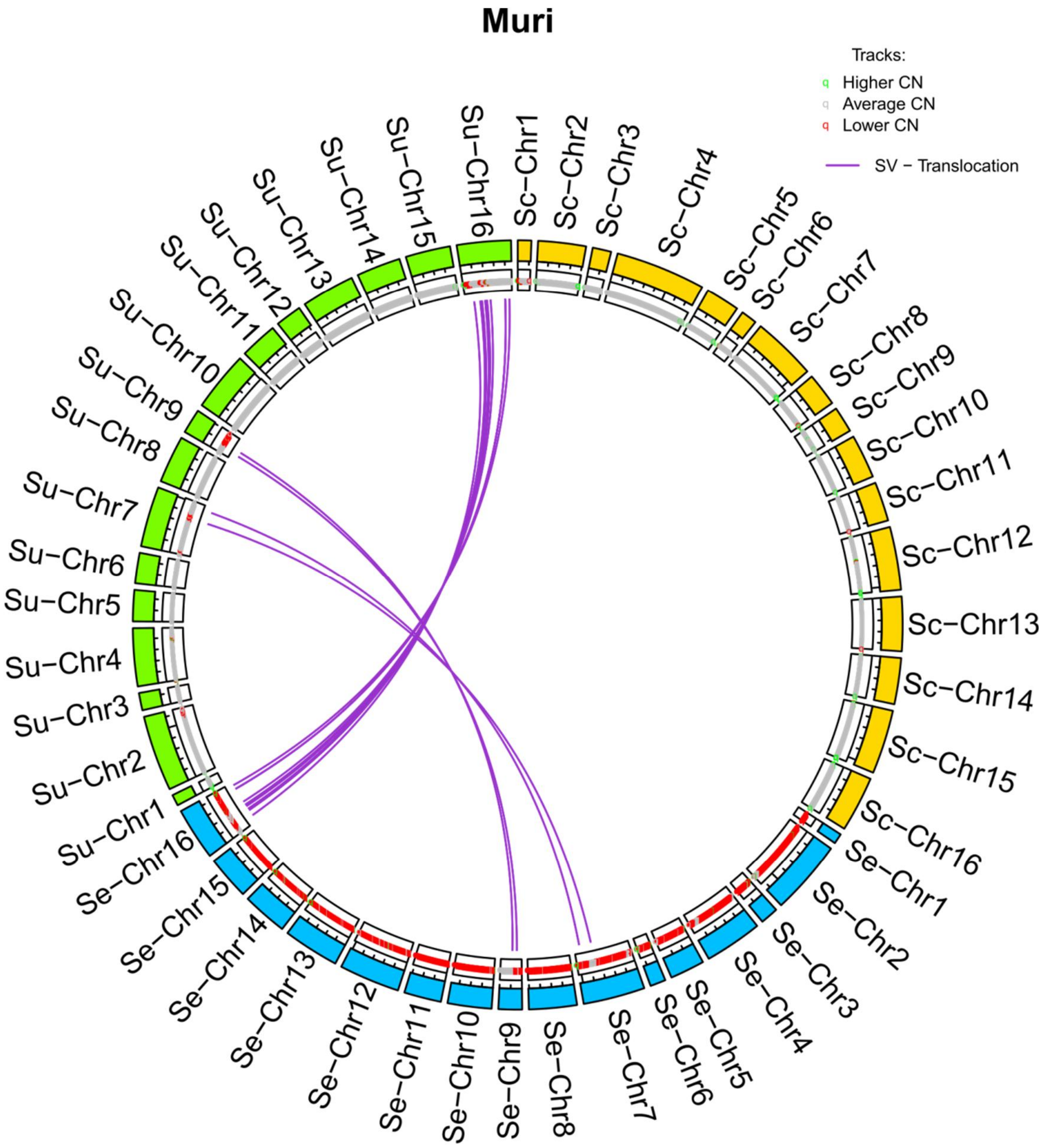

Supplementary Figure S1 – 'Circos-style' plot of Muri's sub-genomes. The outer most track shows the chromosomes of the reference genome to which the sequencing reads were aligned (*S. cerevisiae* chromosomes in yellow, *S. eubayanus* chromosomes in blue, and *S. uvarum* chromosomes in green). The middle track shows the normalized median coverage in 10000 bp windows (green denotes coverage higher than 1.25 times the mean coverage, red denotes coverage lower than 0.75 times the mean coverage, and grey denotes coverage between 0.75 to 1.25 times the mean coverage). Ribbons between chromosomes show interchromosomal translocations detected by Manta. The translocations depict the breakpoints for introgressed regions.

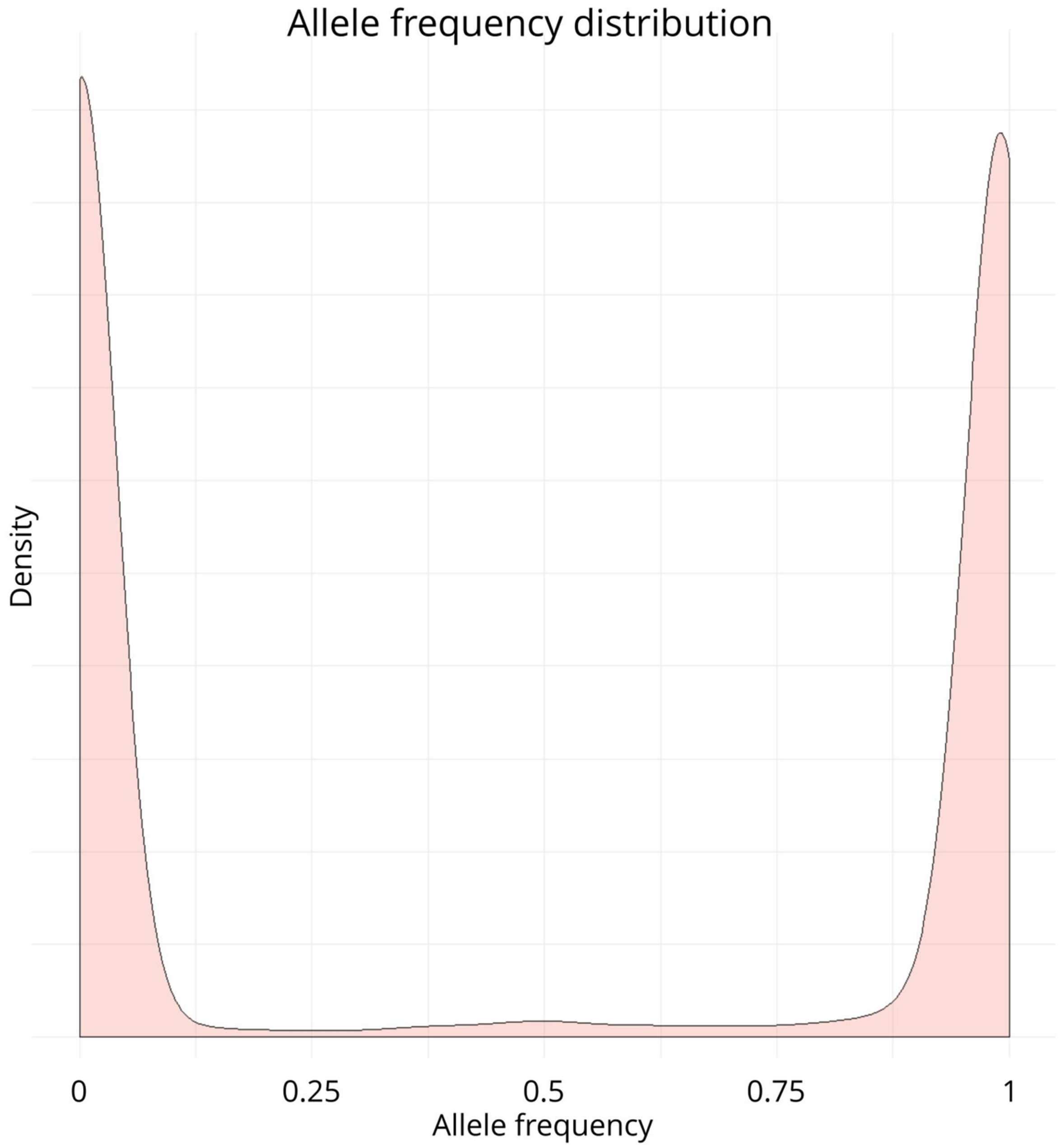

Supplementary Figure S2 – Allele frequency distribution of the single nucleotide polymorphisms detected in the Muri hybrid. Peaks at 0 and 1 suggest a single allele at each site (haploid sub-genomes).

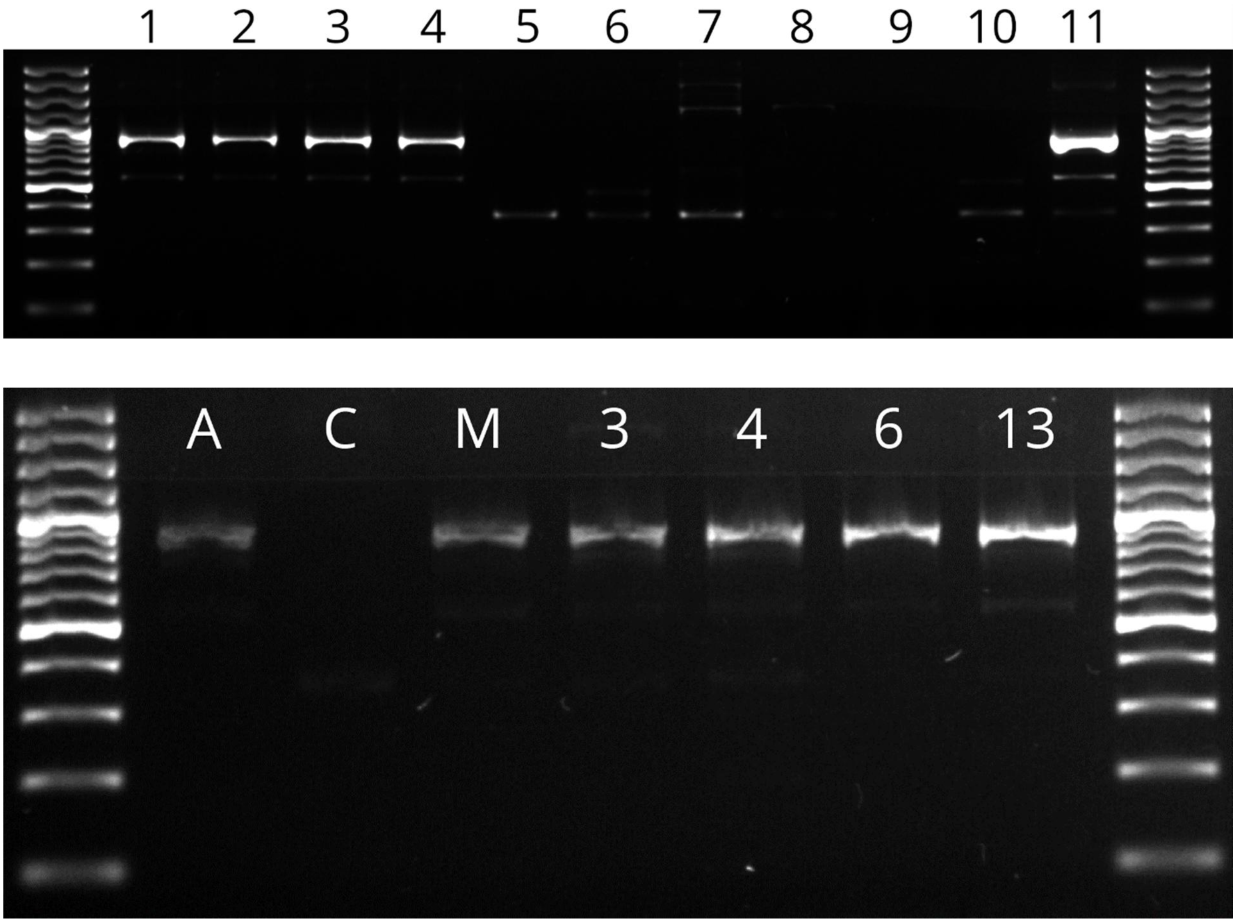

Supplementary Figure S3 – PCR of *STA1* (amplicon size approx. 870 bp) using primers SD-5A and SD-6B (Yamauchi et al., 1998). (**A**) *S. cerevisiae, S. uvarum,* and natural hybrid strains. The lanes are numbered as follows: 1: A240, 2: A241, 3: A242, 4: A243, 5: C992, 6: C993, 7: C995, 8: C996, 9: C997, 10: C998, 11: Muri. (**B**) Muri, *de novo* hybrids, and parent strains. The lanes are numbered as follows: A: A241, C: C995, M: Muri, 3: 3B, 4: 4B, 6: 6A, 13: 13C. A 100 bp ladder was used.

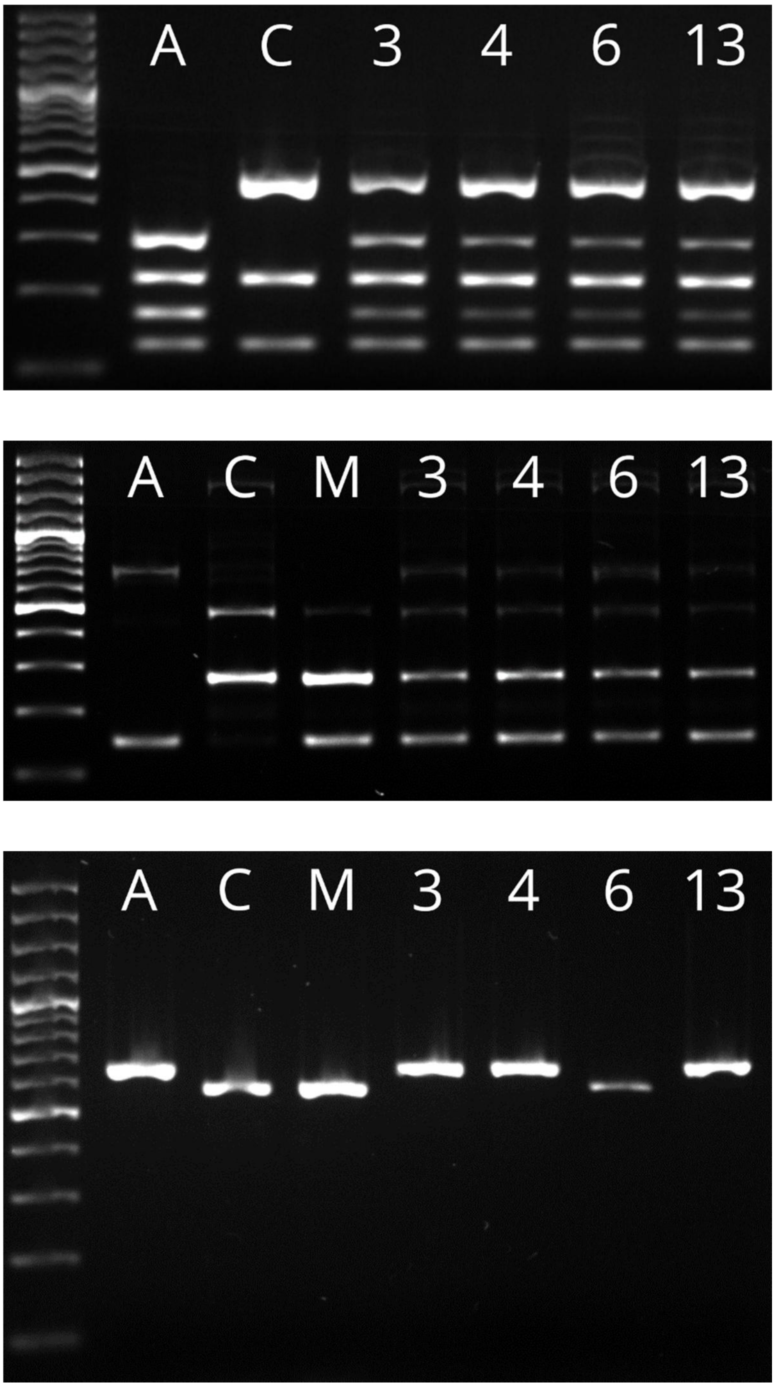

Supplementary Figure S4 – Hybrid confirmation of A241 × C995 *de novo* hybrids. (**A**) ITS-PCR and *HaeIII* digestion. (**B**) PCR using *S. cerevisiae*- and *S. uvarum*-specific primers. (**C**) PCR of the mitochondrial gene *COX2*, followed by *HaeIII* digestion. The lanes are numbered as follows: A: A241, C: C995, M: Muri, 3: 3B, 4: 4B, 6: 6A, 13: 13C. A 100 bp ladder was used.

Supplementary Table S1 – Median sequencing coverage of reads from the Muri hybrid in 10kb windows aligned to the mitochondrial DNA (mtDNA) of *S. cerevisiae* (NCBI-GenBank: NC_001224), *S. eubayanus* (NCBI-GenBank: NW_017264706), and *S. uvarum* (NCBI-GenBank: KX657742).

Reference mtDNA	Start position	End position	Median coverage
S. cerevisiae S288c	0	10000	37
	10000	20000	40
	20000	30000	19638
	30000	40000	40
	40000	50000	40
	50000	60000	2
	60000	70000	15
	70000	80000	4
	80000	85779	5
S. eubayanus FM1318	0	10000	49
	10000	20000	690
	20000	30000	8
	30000	40000	7
	40000	50000	5
	50000	60000	39
	60000	63999	46
S. uvarum CBS395	0	10000	15197
	10000	20000	15545
	20000	30000	17114
	30000	40000	15616
	40000	50000	14490
	50000	60000	14280
	60000	64779	16883

Supplementary Table S2 – Euclidean distance matrix between the strains in the principal component analysis of the dataset with 34 brewing-relevant phenotypic traits (Figure 6).

	**C995**	**Muri**	**3B**	**4B**	**6A**	**13C**
**A241**	10.83	6.38	8.66	7.82	8.33	8.35
**C995**		8.33	5.44	5.53	5.68	6.91
**Muri**			5.81	5.23	6.05	5.75
**3B**				3.05	3.52	4.99
**4B**					1.69	4.48
**6A**						4.53

Supplementary Table S3 – Significantly enriched ($p < 0.05$) gene ontology (GO) terms in lists of genes affected by unique missense or nonsense mutations in Muri (*S. cerevisiae* sub-genome) and A241 (Beer059). Analysis was performed using YeastMine (http://yeastmine.yeastgenome.org). *p*-values are corrected with the Holm-Bonferroni method.

**Muri (*S. cerevisiae* sub-genome)**		
**GO Term**	***p*-Value**	**Number of genes**
biological regulation [GO:0065007]	1.202050e-6	544
regulation of cellular process [GO:0050794]	5.057595e-4	423
regulation of biological process [GO:0050789]	6.117187e-4	448
regulation of response to stimulus [GO:0048583]	0.001424	67
response to stimulus [GO:0050896]	0.006898	339
**A241 (Beer059)**		
**GO Term**	***p*-Value**	**Number of genes**
response to stimulus [GO:0050896]	0.010846	298
response to organic substance [GO:0010033]	0.012249	78

Supplementary Table S4 – The concentrations of aroma compounds (mg/L) and calculated weighting factors based on the aroma thresholds (Meilgaard, 1982) in the beers fermented at 12 °C with the *S. cerevisiae, S. uvarum* and natural hybrid strains.

	Propanol	2-Me-Propanol	2-Me-Butanol	3-Me-Butanol	3-Me-Butyl acetate	Ethyl acetate	Ethyl hexanoate	Ethyl octanoate
**A240**	16.64	11.05	12.08	49.02	0.39	16.89	0.12	0.08
**A241**	16.06	11.50	13.37	37.98	0.55	23.97	0.19	0.09
**A242**	12.90	13.42	13.65	40.29	0.55	18.27	0.16	0.12
**A243**	15.17	13.11	14.13	45.30	0.56	25.43	0.12	0.08
**C992**	8.76	6.70	8.97	22.69	0.12	8.73	0.05	0.00
**C993**	9.86	10.31	12.01	23.39	0.14	14.93	0.05	0.00
**C995**	11.12	9.81	13.06	26.26	0.17	11.91	0.08	0.00
**C996**	21.42	28.69	24.84	57.35	0.61	25.17	0.14	0.05
**C997**	7.21	4.77	5.59	15.33	0.06	2.76	0.00	0.19
**C998**	13.40	13.95	16.62	40.28	0.35	27.73	0.06	0.08
**Muri**	12.17	11.68	13.72	32.19	0.64	32.58	0.17	0.04
**Mean value**	13.15	12.27	13.46	35.46	0.38	18.94	0.10	0.07
**Threshold**	800	200	65	70	1.2	30	0.21	0.9
**Weighting factor**	0.016	0.061	0.207	0.507	0.313	0.631	0.485	0.075

Supplementary Table S5 – The concentrations of aroma compounds (mg/L) and calculated weighting factors based on the aroma thresholds (Meilgaard, 1982) in the beers fermented at 20 °C with the *S. cerevisiae, S. uvarum* and natural hybrid strains.

	Propanol	2-Me-Propanol	2-Me-Butanol	3-Me-Butanol	3-Me-Butyl acetate	Ethyl acetate	Ethyl hexanoate	Ethyl octanoate	Ethyl decanoate	2-Phenylethanol
**A240**	27.81	24.88	23.71	74.26	1.63	39.30	0.42	0.42	0.08	23.64
**A241**	21.23	18.08	21.29	59.39	1.92	53.01	0.34	0.35	0.12	14.27
**A242**	18.56	23.03	22.33	50.53	1.43	41.19	0.44	0.47	0.11	15.46
**A243**	18.59	23.78	23.35	59.14	1.73	45.12	0.41	0.40	0.06	18.67
**C992**	12.22	11.76	14.07	44.42	0.65	20.15	0.08	0.00	0.05	15.23
**C993**	14.24	12.41	14.30	28.54	0.38	18.03	0.00	0.07	0.10	13.82
**C995**	14.14	13.78	15.97	43.78	0.61	20.79	0.10	0.07	0.07	16.08
**C996**	20.82	27.56	24.15	53.98	0.81	26.90	0.13	0.23	0.25	24.08
**C997**	7.83	4.69	5.74	22.06	0.10	3.10	0.08	0.27	0.10	9.71
**C998**	14.13	14.34	15.23	49.98	0.72	21.95	0.00	0.13	0.10	29.49
**Muri**	16.17	16.87	17.45	42.12	0.92	40.53	0.18	0.21	0.16	20.31
**Mean value**	16.88	17.38	17.96	48.02	0.99	30.00	0.20	0.24	0.11	18.25
**Threshold**	800	200	65	70	1.2	30	0.21	0.9	1.5	100
**Weighting factor**	0.021	0.087	0.276	0.686	0.826	1.000	0.943	0.265	0.073	0.182

Supplementary Table S6 – The concentrations of aroma compounds (mg/L) and calculated weighting factors based on the aroma thresholds (Meilgaard, 1982) in the beers fermented at 12 °C with Muri, the *de novo* hybrid, and parent strains.

	Propanol	2-Me-Propanol	2-Me-Butanol	3-Me-Butanol	3-Me-Butyl acetate	Ethyl acetate	Ethyl hexanoate
**A241**	17.39	10.95	13.20	35.87	0.49	28.13	0.21
**C995**	9.65	8.71	11.16	22.29	0.20	10.65	0.10
**Muri**	14.43	13.14	15.36	32.22	0.38	31.65	0.17
**3B**	10.88	8.83	10.05	18.87	0.11	10.29	0.09
**4B**	12.94	11.22	12.31	26.69	0.24	17.39	0.06
**6A**	13.86	10.79	13.73	28.91	0.16	10.07	0.00
**13C**	13.14	11.35	12.21	22.12	0.16	16.90	0.09
**Mean value**	13.18	10.71	12.58	26.71	0.25	17.87	0.10
**Threshold**	800	200	65	70	1.2	30	0.21
**Weighting factor**	0.016	0.054	0.193	0.382	0.209	0.596	0.489

Supplementary Table S7 – The concentrations of aroma compounds (mg/L) and calculated weighting factors based on the aroma thresholds (Meilgaard, 1982) in the beers fermented at 20 °C with Muri, the *de novo* hybrid, and parent strains.

	**Propanol**	**2-Me-Propanol**	**2-Me-Butanol**	**3-Me-Butanol**	**3-Me-Butyl acetate**	**Ethyl acetate**	**Ethyl hexanoate**	**Ethyl octanoate**	**Ethyl decanoate**	**2-Phenylethanol**
**A241**	18.80	15.57	18.62	53.45	0.94	41.54	0.24	0.18	0.07	18.06
**C995**	15.18	15.67	17.72	46.19	0.67	23.41	0.06	0.03	0.04	23.30
**Muri**	17.03	16.99	17.86	42.79	0.78	37.77	0.20	0.13	0.10	23.38
**3B**	18.86	13.91	16.78	37.97	0.45	24.31	0.12	0.12	0.09	21.25
**4B**	21.34	17.74	19.95	53.77	0.86	34.01	0.06	0.09	0.09	26.38
**6A**	24.07	20.26	23.54	56.39	1.08	31.38	0.07	0.06	0.04	27.01
**13C**	25.68	16.96	19.09	40.74	0.80	37.36	0.27	0.11	0.06	19.09
**Mean value**	20.14	16.73	19.08	47.33	0.80	32.83	0.15	0.10	0.07	22.64
**Threshold**	800	200	65	70	1.2	30	0.21	0.9	1.5	100
**Weighting factor**	0.025	0.084	0.294	0.676	0.665	1.094	0.691	0.114	0.046	0.226
